# Galectins at the crossroads of tumor immunity, metabolism, and metastasis: mechanisms, therapeutic resistance, and translational opportunities

**DOI:** 10.3389/fimmu.2026.1849406

**Published:** 2026-07-15

**Authors:** Chengxun Jin, Wenjia Wang, Yihang Gao

**Affiliations:** 1 Department of Otolaryngology, The Second Hospital of Jilin University, Changchun, China; 2 Department of Laboratory Medicine, The Second Hospital of Jilin University, Changchun, China

**Keywords:** galectins, immune evasion, metabolic reprogramming, therapeutic resistance, tumor microenvironment

## Abstract

Galectins, a structurally conserved family of β-galactoside-binding lectins, have emerged as key regulators of cancer progression. However, their integrated roles across tumor immunity, metabolic rewiring, and metastasis have yet to be fully defined. In this review, we synthesize current evidence on major galectin family members, particularly galectin (Gal)-1, Gal-3, Gal-4, Gal-7, Gal-9, and Gal-13, and delineate how they drive tumor progression through mechanistically distinct yet convergent pathways. Specifically, galectins induce T-cell dysfunction through T-cell immunoglobulin and mucin-domain containing-3 (TIM-3)- and programmed cell death protein 1 (PD-1)-associated signaling, reprogram macrophages and myeloid-derived suppressor cells (MDSCs) via phosphoinositide 3-kinase/protein kinase B (PI3K/AKT), Janus kinase/signal transducer and activator of transcription (JAK/STAT), and nuclear factor kappa B (NF-κB) pathways, and modulate innate immune effectors, including natural killer (NK) cells, neutrophils, and dendritic cells, in a context-dependent manner. Beyond immune regulation, galectins reshape tumor metabolism through effects on glycolysis, lipid metabolism, and glycan remodeling, and differentially regulate ferroptosis susceptibility, with the contrasting roles of Gal-1 and Gal-13 underscoring functional diversity within the family. Emerging evidence further implicates galectins in resistance to chemotherapy, targeted therapy, immune checkpoint blockade, and chimeric antigen receptor T-cell (CAR-T) therapy, positioning them as candidate therapeutic targets. We also discuss galectin-directed strategies, including small-molecule inhibitors, nanomedicine-based platforms, vaccination approaches, and rational combination therapies, and highlight the promise of multi-galectin biomarker panels for precision oncology.

## 1 Introduction

Cancer remains a leading cause of mortality worldwide, and immune evasion together with therapeutic resistance continues to represent major barriers to effective treatment (1–3). Although immunological and oncogenic signaling pathways have been extensively studied, accumulating evidence suggests that glycan signaling is an important yet underappreciated regulator of the tumor–immune axis (4, 5). Within the complex TME, galectins, a family of evolutionarily conserved β-galactoside-binding lectins, have emerged as key mediators linking glycan signaling to immune and metabolic adaptation (6–8). As a structurally compact family of only ~15 members, galectins nonetheless exert remarkably diverse functions through both carbohydrate-dependent interactions with extracellular glycoconjugates and carbohydrate-independent interactions with cytosolic and nuclear targets (9).

Although galectins were initially characterized as extracellular glycan readers involved in cell adhesion, migration, and apoptosis, they are now recognized as multifunctional regulators of tumor progression through their effects on immune checkpoint signaling, myeloid cell polarization, stromal remodeling, and metabolic reprogramming (10–16). Distinct galectin family members integrate intracellular signaling pathways with extracellular matrix organization and immune checkpoint regulation, thereby coordinating tumor adaptation to environmental stresses such as hypoxia, nutrient deprivation, and inflammatory signaling (7, 17, 18). Dysregulated galectin expression has been linked to tumor progression, metastatic dissemination, and resistance to chemotherapy, radiotherapy, immunotherapy, and adoptive T-cell therapy (17, 19–23). Importantly, galectins function as molecular hubs within the TME, coordinating interactions among tumor cells, cancer-associated fibroblasts (CAFs), endothelial cells, and diverse immune cell populations. Gal-1 and Gal-3 have been shown to reprogram tumor-associated macrophages and endothelial cells toward immunosuppressive phenotypes. Gal-9 further contributes to immunosuppression through its interactions with inhibitory receptors such as TIM-3 and PD-1, thereby promoting T-cell exhaustion and supporting regulatory T-cell maintenance (24–26).

In this Review, we synthesize current mechanistic and translational insights into how galectins coordinate immunosuppression and metabolic adaptation within the TME. We focus on emerging concepts in galectin-driven immune–metabolic crosstalk, their roles in resistance to conventional and immune-based therapies, and therapeutic opportunities arising from galectin-targeted interventions. By positioning galectins as integrative regulators rather than isolated lectins, this Review aims to provide a conceptual framework for targeting glycan-mediated pathways to overcome immune resistance and improve cancer treatment outcomes.

## 2 Overview of the galectin family: structure, biology, and function

Galectins are an evolutionarily conserved family of β-galactoside-binding lectins that recognize glycan structures on glycoproteins and glycolipids. They regulate a broad range of biological processes, including cell signaling, proliferation, apoptosis, adhesion, migration, and disease pathogenesis (27–32). Structurally, galectins contain either one carbohydrate recognition domain (CRD) or two distinct CRDs, each with affinity for β-galactoside-containing glycans (33). Galectins are structurally classified into three groups: proto-type galectins, which contain a single CRD; tandem-repeat galectins, which contain two distinct CRDs connected by a linker peptide; and the chimera-type galectin, Gal-3, which contains a single CRD and a unique N-terminal domain (33, 34). The chimera-type galectin, Gal-3, is distinguished by a single CRD linked to an extended N-terminal domain containing multiple proline-, glycine-, and tyrosine-rich repeats (27).

Galectins recognize β-galactoside-containing glycoconjugates at the cell surface, within the extracellular matrix, and in intracellular compartments (32). Although galectins are synthesized in the cytosol, they lack canonical signal peptides and are secreted through non-classical pathways (32, 35). Galectin expression is tissue-specific and developmentally regulated: Gal-1 is highly expressed in immune cells, Gal-3 in epithelial cells, and Gal-9 in the thymus (14, 36, 37). Functionally, galectins act both intracellularly, where they interact with signaling and survival-associated proteins such as Ras and Bcl-2, and extracellularly, where they bind glycoconjugates on integrins, growth factor receptors, and pathogens (38–41).

Their ability to engage in multivalent interactions enables galectins to organize glycan-dependent signaling networks that influence cell fate, survival, migration, and immune function. In cancer, galectins contribute to multiple hallmarks of disease, including malignant transformation, angiogenesis, immune evasion, invasion, and metastasis. Accordingly, galectins have emerged as promising therapeutic targets in cancer (28). Galectins also regulate immune responses and, in some contexts, function as pattern-recognition receptors. For example, Gal-3 can bind microbial glycans and contribute to innate host defense, whereas Gal-1 and Gal-9 modulate T-cell responses and immune tolerance through interactions with inhibitory receptors such as TIM-3 (42–46). At the cell surface, galectins promote glycan lattice formation, thereby stabilizing receptors such as EGFR and enhancing signaling pathways involved in proliferation and angiogenesis (12, 47). Representative tumor-promoting mechanisms include Gal-3-mediated suppression of apoptosis through interactions with Bax, Gal-1-driven immune evasion via induction of T-cell apoptosis, and Gal-9-associated recruitment of myeloid-derived suppressor cells (MDSCs) (48–51). The therapeutic relevance of galectins is further supported by the development of galectin-targeting strategies, including Gal-3 inhibitors such as TD139, which have shown promise in inflammatory and neoplastic settings. By decoding glycosylation-dependent signals in both extracellular and intracellular contexts, galectins serve as versatile regulators of cell communication and tissue adaptation.

## 3 Galectin-mediated immune evasion

This section examines the multifaceted roles of galectins in tumor immune evasion, organized across three complementary axes. We first address how galectins engage and modulate immune checkpoint pathways to suppress adaptive immunity, then explore their capacity to reprogram myeloid cell populations within the TME, and finally consider their regulation of innate effector cells including NK cells, neutrophils, and dendritic cells (**Table 1**).

**Table 1:** Role of galectins in tumor immune evasion, immunotherapy resistance, and tumor microenvironment regulation.

Galectin/molecule	Cancer type	Study design	Key mechanism	Immunological effect & therapeutic implication	Ref.
Galectin-1	Head & Neck Cancer	*In vitro*; *In vivo*; Clinical (ICI-treated)	Gal-1 reprograms tumor endothelium to upregulate PD-L1/Gal-9, forming an immunosuppressive vascular barrier.	T cell exclusion; inverse correlation with ICI response. Gal-1 blockade synergizes with anti-PD-1 ± RT.	(52)
	Colorectal Cancer	*In vivo* (Lgals1−/−); Bioinformatics (TCGA)	Gal-1 expands CD8^+^CD122^+^PD-1^+^ Tregs and enhances their suppressive capacity.	Elevated CD8^+^ Treg frequency; reduced anti-tumor immunity; high Gal-1/CD8^+^ Treg signature → poor CRC prognosis.	(53)
	Lung, Melanoma	*In vitro*; *In vivo* (murine)	KLF12 transcriptionally represses Gal-1; low KLF12 → elevated Gal-1 → impaired CD8^+^ T cell infiltration.	Suppressed CD8^+^ T cell function; low KLF12 correlates with anti-PD-1 resistance. KLF12/Gal-1 targeting restores ICI efficacy.	(54)
	HCC	Clinical; *In vitro*; *In vivo* (Foxp3^DTR^)	Tumor Gal-1 induces TAM CCL20 upregulation via PI3K/AKT/NF-κB; CCL20 recruits CCR6^+^Foxp3^+^ Tregs.	Treg accumulation suppresses CD8^+^ T cells; anti-PD-1 resistance. Gal-1 inhibition restores anti-PD-1 efficacy.	(24)
	HCC	*In vitro*; *In vivo* (murine)	TLR2-dependent secretory autophagy drives TAM Gal-1 secretion via Rab11/VAMP7 vesicle trafficking.	Elevated systemic/intratumoral Gal-1 promotes HCC growth; TAM autophagy-derived Gal-1 correlates with poor OS/PFS.	(55)
	Multiple solid tumors	*In vitro* (proteomics; 3D model)	Epithelial/stromal Gal-1 induces TAM phenotype via JAK/STAT, upregulating PD-L1 and IDO1.	TAMs acquire dual immunosuppressive axis (PD-L1 + IDO1); T cell suppression.	(56)
	Breast Cancer	scRNA-seq; *In vitro*; *In vivo* (syngeneic)	TAM-derived Gal-1 mediates early immunosuppressive TME programming; TAM depletion reduces Gal-1.	Gal-1 suppresses CD8^+^ T cells and drives EMT. Gal-1 inhibition reinvigorates CD8^+^ T cells and potentiates ICI.	(57)
	Head & Neck/Lung	*In vivo* (murine); RNA-seq	Gal-1 stabilizes STING → NF-κB activation → CXCL2-driven PMN-MDSC expansion and premetastatic niche formation.	PMN-MDSC accumulation facilitates ECM remodeling and metastasis; Gal-1 acts as paradoxically protumoral STING regulator.	(58)
	Lung Cancer	*In vitro*; *In vivo*; Clinical IHC	Gal-1 upregulates CAF TDO2 via AKT; CAF-derived kynurenine inhibits DC differentiation and promotes migration via AKT/WNK1.	Impaired DC/T cell responses; enhanced EMT and metastasis. TDO2 inhibitor rescues DC/T cell function *in vivo*.	(59)
	Glioma	*In vitro*; *In vivo* (intracranial)	Gal-1 suppresses miR-1983–TLR7–IFN-β innate circuit; exosomal miR-1983 normally activates TLR7 in pDCs → IFN-β → NK activity.	Gal-1 inhibits NK-mediated glioma killing by blocking innate IFN-β. Anti-Gal-1 activates this circuit 10–14 days before adaptive immunity.	(60)
Galectin-3	Lung Cancer	scRNA-seq; *In vitro*; *In vivo* (TREM2 cKO); MS	Gal-3 is a TREM2 ligand; Gal-3–TREM2 interaction impairs phagocytosis and converts TREM2^+^ macrophages to immunosuppressive TAMs via CCL2-CCR2.	Attenuated antigen presentation; immunosuppressive myeloid TME. Combined Gal-3 (GB1107) + TREM2 blockade inhibits progression.	(61)
	GBM; Breast Brain Met.	*In vitro*; *In vivo* (brain tumor models)	Gal-3 in microglia/TAMs promotes cancer migration and immunosuppressive microglial activation; depletion triggers IFN responses.	Gal-3-driven immunosuppressive microglial phenotype supports brain tumor growth. Depletion restores anti-tumor IFN responses.	(62)
	Breast Cancer	*In vitro*; *In vivo* (syngeneic)	Hypoxia induces TAM Gal-3 synthesis/secretion via NF-κB/ROS; elevated Gal-3 promotes tumor growth and metastasis.	Hypoxia-driven TAM Gal-3 creates pro-metastatic TME. Gal-3 inhibitor (MCP) + macrophage depletion ± anti-angiogenics synergize.	(63)
	Multiple (allograft)	*In vivo* (Gal3−/− host mice)	Tumor-derived Gal-3 gradient guides M2 macrophages (low Gal-3-expressing) into tumor.	Enhanced M2 TAM infiltration and angiogenesis. Gal-3 gradient drives pro-tumoral M2 recruitment.	(64)
	HCC	*In vitro* (RIP, ChIP); *In vivo*	M2-TAM exosomal lncRNA NEAT1 recruits KLF5 to LGALS3 promoter, upregulating Gal-3 in HCC cells.	Gal-3 depletes perforin^+^CD8^+^ T cells → immune escape. NEAT1 silencing reverses Gal-3 upregulation and restores surveillance.	(65)
	Ovarian Cancer	*In vitro*; *In vivo* (SCID xenograft)	OC-derived exosomal Gal-3 activates β-catenin → M2 macrophage polarization. Shikonin reduces exosomal Gal-3.	Exosomal Gal-3/β-catenin-driven M2 polarization promotes invasion/immunosuppression. Shikonin blocks this axis.	(66)
	Cervical Cancer	Clinical; *In vitro*; *In vivo*	GAL3 promotes MDSC recruitment via STAT3/Akt; MDSCs inhibit CD8^+^ T cells through IL-6 and CXCL2.	GAL3 correlates with LN metastasis, recurrence, poor survival. GAL3 + MDSC inhibitors reduce tumor burden *in vivo*.	(67)
	Ovarian Cancer (HGSC)	Clinical (ascites); *In vitro*	Elevated ascites Gal-3 induces ROS production in tumor-associated neutrophils.	Neutrophil-derived ROS decreases NK viability and cytotoxicity. Gal-3 targeting may restore NK function in HGSC TME.	(68)
Galectin-4	Multiple; LCMV	*In vitro*; *In vivo* (Gal-4 KO; syngeneic)	Gal-4 is required for adequate MHC-I surface expression on DCs; deficiency impairs DC antigen presentation → deficient CD8^+^ T cell priming.	Gal-4 KO → enhanced tumor growth and impaired CD8^+^ T cell responses. Exogenous Gal-4 enhances cancer vaccine and PD-1 blockade efficacy.	(69)
	Colorectal Cancer	Bioinformatics; *In vitro*; *In vivo*	TP53 mutations downregulate LGALS4; LGALS4 activates CCL4/CCR5 via NF-κB, promoting immune cell recruitment.	LGALS4 loss drives immune exclusion in CRC. LGALS4 overexpression improves infiltration and synergizes with anti-PD-L1.	(70)
Galectin-7	Esophageal Cancer	*In vitro*; *In vivo* (PD-1 KO); Clinical IHC	Gal-7 binds PD-1 N-glycosylation sites (N74/N116), recruits SHP-2 → NFAT suppression. Fully PD-1-dependent.	Reduced CD4^+^ T cell frequency *in vitro*/*in vivo*. Novel PD-1 glycosylation-dependent immunosuppressive mechanism.	(71)
Galectin-9	Multiple cancers	*In vitro*; *In vivo*; Bioinformatics	PD-1 directly binds Gal-9 (TIM-3 ligand), attenuating Gal-9/TIM-3-induced T cell death; sustains PD-1^+^TIM-3^+^ exhausted T cell survival. IFN-β/γ promotes Gal-9 secretion.	PD-1 protects exhausted T cells from Gal-9/TIM-3 apoptosis; high Gal-9 → poor prognosis. Anti-Gal-9 + anti-GITR synergize.	(26)
	Gastric Cancer	Bioinformatics (TCGA-STAD); Ex vivo	Gal-9/TIM-3 promotes Treg suppression and CD8^+^ T cell dysfunction independently of PD-1/PD-L1.	PD-1-independent immune evasion; CD8^+^ exhaustion and Treg expansion → poor survival. Gal-9 as anti-PD-1 resistance biomarker.	(45)
	CLL	Clinical (RT-PCR; 25 CLL vs. 15 controls)	Gal-9 and PD-L1 mRNA overexpressed in CLL cells (p<0.0001 and p=0.005); expression increases with Rai stage.	Concurrent Gal-9/TIM-3 and PD-1/PD-L1 activation in CLL. Gal-9 and PD-L1 as prognostic biomarkers for progression.	(72)
	Colorectal Cancer	Clinical; Bioinformatics (GSEA)	TIM-3/Gal-9 elevated in CRC vs. non-tumor; TIM-3 higher in PIK3CA-mutant tumors. High expression downregulates IFN/TNF-α/NF-κB signatures.	Immune suppression and cell cycle upregulation; immunoevasive phenotype in PIK3CA-mutant CRC.	(73)
	Thyroid Cancer	*In vitro*; *In vivo* (murine)	TC-derived SCF recruits mast cells; intratumoral mast cells express elevated Gal-9 → suppress CD8^+^ T cell immunity.	Progressive mast cell/Gal-9-mediated CD8^+^ suppression with tumor stage. Blocking mast cell Gal-9 restores CD8^+^ function.	(74)
	Gastric Cancer	*In vitro*; *In vivo*; miRNA analysis	LGALS9 overexpressed in GC; reduces CD8^+^ T cell infiltration. miR-491-5p directly targets LGALS9; KO reverses immunosuppression.	miR-491-5p/LGALS9 axis regulates CD8^+^ immunity. LGALS9 inhibition (genetic/miRNA) is a promising GC immunotherapy strategy.	(75)
	Multiple solid tumors	*In vitro*; *In vivo* (murine)	T cells induce cancer cell Gal-9 secretion (shedding or autophagy-dependent); secreted Gal-9 cooperates with VISTA to attenuate T cell activity.	Self-reinforcing immunosuppressive loop. Gal-9/VISTA cooperative blockade proposed as combination immunotherapy.	(76)
	VASTs	Phase II trial (LATENT; n=40; avelumab)	Gal-9 upregulated on T cells in VASTs; co-expression with PD-1/TIGIT synergistically enhances exhaustion. Persistent TCR stimulation drives membrane translocation.	T cell Gal-9 is a novel exhaustion marker; high Gal-9 in TME predicts poor anti-PD-L1 response. First clinical report.	(77)
	HCC	Clinical IHC (TMA; n=94 + 60); Multivariate	Gal-9 in 79% HCC; low Gal-9 + low PD-L1 + low CD8^+^TIL independently predicts extremely poor survival (HR 0.29; p<0.001).	Low checkpoint expression signals immune-cold phenotype → worst prognosis. Combined biomarker panel superior to individual markers.	(78)
	Glioma (1p/19q codel.)	Bioinformatics; *In vitro*; *In vivo*; IHC	1p/19q codeletion reduces C/EBPα → downregulates Gal-9/TIM-3; 19q loss is primary driver. Validated in 3 datasets.	1p/19q codeletion promotes antitumor immunity via Gal-9/TIM-3 downregulation. 1p/19q as biomarker for TIM-3-targeted therapy.	(79)
	Nasopharyngeal Carcinoma	*In vitro*; *In vivo* (adoptive CTL)	Cell-associated Gal-9 on tumor membrane interacts with CTLs; promotes tumor autophagy and restricts necrosis upon CTL contact.	Tumor evasion via Gal-9-induced autophagy. Autophagy inhibition + Gal-9 KD restores CTL-mediated tumor control.	(80)
	Colon Cancer	*In vitro* (THP-1; co-culture)	Gal-9 binds TIM-3, recruits PI3K-p85 → PI3K/Akt → M2 polarization (high arginase-1, CD163, IL-10; low iNOS).	M2 polarization increases cancer viability, migration, invasion. TIM-3 or PI3K inhibition blocks this axis.	(81)
	Muscle-Invasive Bladder	Clinical IHC (TMA n=141); Cox regression	Gal-9^+^TAM frequency increases with stage/grade; correlates with Tregs, mast cells, PD-1^+^TIM-3^+^CD8^+^ exhaustion.	Gal-9^+^TAMs independently predict poor OS/RFS. High Gal-9^+^TAM identifies favorable adjuvant platinum chemo responders.	(82)
	Pan-cancer (LN Met.)	scRNA-seq (11 cancers; 859K cells); *In vivo*	Gal-9^HI^ macrophages enriched in LN metastases vs. primary tumors; Gal-9/BTK/STAT3 → IL-10 → immune-tolerant LN niche.	LN TME more immunosuppressive than primary. STAT3 inhibition + PD-1 blockade depletes Gal-9^HI^ macrophages and reduces LN metastasis.	(58)
	Metastatic Melanoma	Clinical (plasma/IHC); *In vitro* (PBMC)	Gal-9 elevated 3.6-fold in plasma; induces Th1 apoptosis, Th2-biased cytokines, and M2 (CD206^+^) differentiation.	Systemic Th2 polarization and reduced 2-year survival. Gal-9 as prognostic biomarker and therapeutic target.	(83)
	NPC	*In vitro* (EV/DC/T cell assays)	NPC-derived small EVs carry surface Gal-9; vesicular Gal-9 induces mregDCs with oxidative metabolism and high IL-4.	Gal-9-carrying EVs inhibit T cell proliferation via mregDCs induction. Anti-Gal-9 abrogates EV-mediated tolerization.	(84)
	Multiple epithelial cancers	*In vitro* (co-culture; cytotoxicity)	Gal-9 externalizes PS and downregulates CD47 on cancer cells; simultaneously activates neutrophils (adhesion, granule mobilization).	Activated neutrophils perform trogocytosis and augmented ADCP. Gal-9 triggers potent neutrophil-mediated innate anti-cancer immunity.	(85)
	Glioma (IDH-stratified)	scRNA-seq (18 pts); Ex vivo; Spectral cytometry; IHC	Gal-9 abundant in glioma-associated microglia (not tumor cells); Gal-9^+^ microglia upregulate adhesion/phagocytosis genes. KD impairs MG–GSC adhesion/phagocytosis.	Non-canonical pro-phagocytic role of microglial Gal-9 (opposite to T cell-suppressive role). Harnessable for GBM immunotherapy.	(86)
	GBM	*In vitro*; *In vivo*; Bioinformatics; HIF-2α KO	Gal-9 highest in microglia/macrophages at hypoxic tumor edge; regulated by HIF-2α. Gal-9^HI^ MG: inflammatory but less phagocytic; Gal-9^Low^: superior phagocytosis.	Functional dichotomy in microglial Gal-9 subsets under hypoxia. Gal-9 blockade suppresses tumor growth; HIF-2α/Gal-9 axis as novel GBM target.	(87)
TIM-3/ATXN3	Melanoma	*In vitro*; *In vivo* (immunocompetent + T cell-deficient)	Tumor-intrinsic TIM-3 is growth-suppressive; TIM-3 blockade activates MAPK in tumor cells → growth in T cell-deficient hosts; anti-tumor in T cell-competent hosts.	Dual opposing cell-type-specific TIM-3 roles. MAPK inhibition reverses tumor-promoting effects; combined TIM-3 + MAPK inhibition maximizes efficacy.	(88)
	Lung Adenocarcinoma; Melanoma	CRISPR screen; *In vitro*; *In vivo*; Bioinformatics	ATXN3 (deubiquitinase) identified as positive PD-L1 transcriptional regulator; protects IRF1, STAT3, HIF-2α from degradation under IFN-γ/hypoxia.	ATXN3-mediated PD-L1 upregulation drives immune evasion. ATXN3 deletion abolishes IFN-γ/hypoxia-induced PD-L1 and sensitizes to ICI.	(89)

### 3.1 Galectins and associated checkpoint regulators in tumor immune evasion

Among the immunosuppressive mechanisms operating within the tumor microenvironment (TME), members of the galectin family have emerged as important modulators of immune checkpoint signaling. Gal-9, the canonical ligand for TIM-3, is one of the best-characterized components of this network. Yang et al. demonstrated that PD-1 itself binds Gal-9, attenuating Gal-9/TIM-3-mediated T cell death and sustaining PD-1^+^TIM-3^+^ exhausted T cell persistence; anti-Gal-9 therapy selectively expanded intratumoral TIM-3^+^ CD8^+^ T cells, and its combination with a Treg-depleting GITR agonist produced synergistic anti-tumor activity (26). These findings position Gal-9 as a potential coordinator of crosstalk between two co-expressed inhibitory pathways, with possible implications for combination immunotherapy design.

The clinical relevance of the Gal-9/TIM-3 axis has been corroborated across multiple tumor types. In gastric cancer, LGALS9 and HAVCR2 are upregulated in tumor tissue and independently associated with poor patient survival; Gal-9 promotes CD8^+^ T cell dysfunction and Treg suppressive activity through TIM-3 engagement independently of PD-1 signaling, identifying this axis as a plausible driver of anti-PD-1 resistance (45). A post-transcriptional regulatory layer was identified by Huang et al., showing that miR-491-5p suppresses LGALS9, restores CD8^+^ T cell cytotoxicity and reduces tumor growth, with effects comparable to those of LGALS9 genetic knockout (75). In colorectal cancer (CRC), TIM-3 and Gal-9 protein levels are significantly elevated in tumor versus adjacent tissue; TIM-3 is particularly high in PIK3CA-mutant tumors and correlates with IL-10, IL-17, and chemokine signatures reflecting a broadly immunoreactive transcriptional state (73). In chronic lymphocytic leukemia, both Gal-9 and PD-L1 mRNA are overexpressed relative to healthy controls and rise with advancing Rai stage, marking them as candidate prognostic biomarkers (72). In gliomas, the transcription factor C/EBPα governs Gal-9 and TIM-3 co-expression; chromosome 1p/19q codeletion reduces C/EBPα activity, downregulating both molecules and enhancing anti-tumor immunity across three independent patient cohorts (79).

An important exception to the uniformly immunosuppressive interpretation of Gal-9 arises in hepatocellular carcinoma (HCC), where low intratumoral Gal-9, together with low PD-L1 and low CD8^+^ TIL counts, predicted markedly worse HCC-specific survival (hazard ratio 0.29 for the combined marker set) in two independent cohorts (78). This apparent paradox may reflect an immune-excluded (“immune desert”) phenotype rather than active checkpoint-mediated suppression, and cautions against applying galectin-based biomarkers uniformly across tumor types.

The cellular sources and mechanisms of tumor-associated Gal-9 are contextually diverse. In thyroid cancer, tumor-associated mast cells recruited by tumor-derived stem cell factor expressed elevated Gal-9 and suppressed CD8^+^ T cell immunity *in vitro*; Gal-9 blockade on these cells reversed immunosuppression (74). Schlichtner et al. identified a distinct induction mechanism in solid tumors: T lymphocytes themselves trigger Gal-9 secretion from otherwise Gal-9-quiescent tumor cells via proteolytic shedding and autophagy-dependent lysosomal release, with the secreted protein subsequently cooperating with VISTA to attenuate T cell activity (76). In nasopharyngeal carcinoma (NPC), membrane-anchored Gal-9 on tumor cells interacted with infiltrating cytotoxic T lymphocytes to promote tumor cell autophagy rather than necrosis, conferring resistance to CTL-mediated killing; autophagy inhibition reversed this resistance, and Gal-9 knockdown enhanced tumor suppression upon adoptive CTL transfer (80). In patients with virus-associated solid tumors, Gal-9 upregulation on T cells correlated with dysfunctional effector phenotypes; co-expression with PD-1 or TIGIT synergistically amplified T cell exhaustion, and higher Gal-9 mRNA in the TME was associated with poorer response to anti-PD-L1 therapy (77).

Other galectin family members engage immune checkpoints through mechanistically distinct routes. Galectin-7 reduced CD4^+^ T cell frequency in a strictly PD-1-dependent manner by binding N-glycosylation sites N74 and N116 on PD-1, recruiting SHP-2 and suppressing NFAT activity, an effect absent in PD-1 knockout mice (71). By contrast, galectin-4 exerts immune-stimulatory effects: Gal-4 deficiency impaired antigen–MHC-I complex display on dendritic cells, compromising CD8^+^ T cell responses; systemic Gal-4 administration enhanced cancer vaccine efficacy and PD-1 blockade responses *in vivo* (69). Consistent with a tumor-suppressive role, TP53 mutations in CRC drive LGALS4 downregulation, and LGALS4 overexpression activated the CCL4/CCR5/NF-κB axis to recruit immune cells and synergized with PD-L1 blockade (70), highlighting functional heterogeneity within the galectin family.

Gal-1 contributes an additional immune evasion layer. In head and neck cancer, tumor-secreted Gal-1 reprogrammed the tumor endothelium to upregulate surface PD-L1 and Gal-9, converting the vasculature into a T cell-exclusion barrier; Gal-1 blockade increased intratumoral T cell infiltration and synergized with anti-PD-1 therapy (52). In CRC, Gal-1 expanded CD8^+^CD122^+^PD-1^+^ regulatory T cells, and TCGA analysis showed that high Gal-1 with elevated CD8^+^ Treg score defined a poor-prognosis subgroup (53). The transcription factor KLF12 directly represses the LGALS1 promoter; low tumor KLF12 derepresses Gal-1, suppresses CD8^+^ T cell infiltration, and confers anti-PD-1 resistance; KLF12 restoration or Gal-1 inhibition reversed these effects across multiple mouse tumor models (54).

Two complementary regulators extend this immune evasion network further. Tim-3 on melanoma cells functions as a cell-intrinsic tumor suppressor: antibody-mediated Tim-3 blockade promoted MAPK-dependent tumor growth in T cell-deficient mice while enhancing anti-tumor immunity in T cell-competent hosts, a dual-context effect pharmacologically resolvable by concurrent MAPK inhibition (88). At the transcriptional level, a CRISPR screen identified the deubiquitinase ATXN3 as a positive regulator of PD-L1 transcription downstream of IFN-γ and hypoxia; ATXN3 stabilized IRF1, STAT3, and HIF-2α, and its deletion abolished inducible PD-L1 expression and improved checkpoint blockade efficacy preclinically (89).

**Figure 1:**
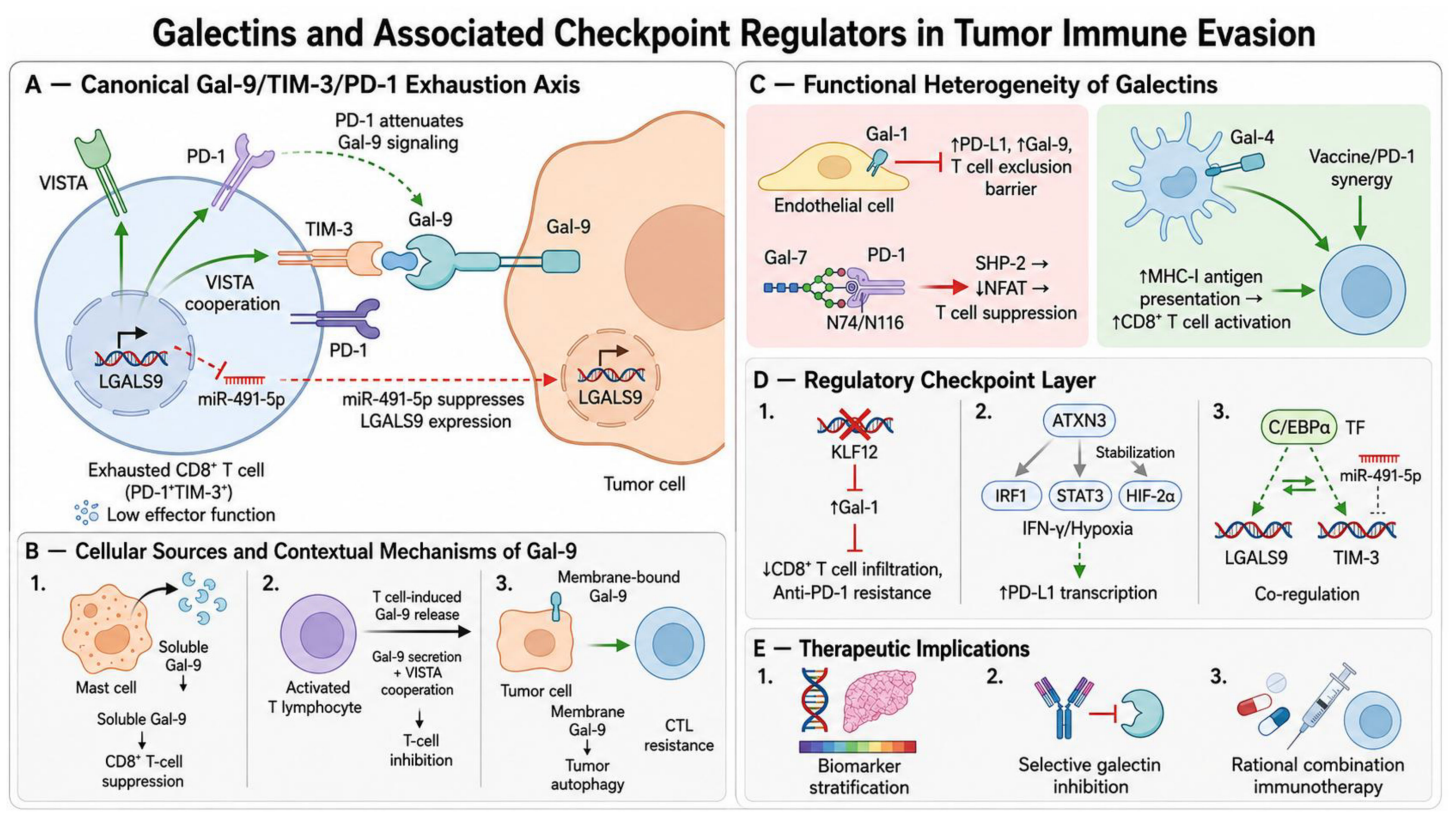
Galectins and associated checkpoint regulators in tumor immune evasion. **(A, B)** Gal-9 is the canonical TIM-3 ligand; it binds PD-1 to attenuate T-cell death, sustains exhausted PD-1^+^TIM-3^+^ CD8^+^ T cells, and contributes to Treg-mediated suppression and anti-PD-1 resistance. In addition, mast cell-derived soluble Gal-9 suppresses CD8^+^ T-cell effector function, activated T lymphocytes induce Gal-9 release from tumor cells that cooperates with VISTA to attenuate T-cell activity, and membrane-bound Gal-9 on tumor cells promotes autophagy-mediated resistance to cytotoxic T lymphocyte (CTL)-mediated killing. **(C–E)** Gal-1 and Gal-7 exert immunosuppressive effects through endothelial reprogramming and PD-1/SHP-2/NFAT signaling, whereas Gal-4 enhances anti-tumor immunity by promoting MHC-I antigen presentation. KLF12, ATXN3, and C/EBPα further regulate this checkpoint network, highlighting the therapeutic potential of selective galectin inhibitors and rational combination immunotherapy.

Collectively, these studies support a multi-layered regulatory network in which galectins—Gal-9, Gal-1, and Gal-7, alongside the immune-stimulatory effects described for Gal-4, interact with canonical checkpoint molecules to shape anti-tumor immunity across diverse cancer types. A recurring finding is that some galectin-driven suppressive mechanisms may not be fully addressed by current PD-1/PD-L1 monotherapy. Priority research directions include clinical evaluation of selective galectin inhibitors, tumor-type-stratified biomarker profiling, and mechanistic dissection of galectin crosstalk with non-canonical regulators such as ATXN3 to guide rational combination strategies (**Figure 1**).

### 3.2 Galectins as orchestrators of myeloid cell reprogramming in the tumor microenvironment

Beyond their direct effects on T cell checkpoint signaling described in section 4.1, galectins also contribute to immune regulation by reprogramming tumor-associated myeloid cells. Tumor-associated macrophages (TAMs), myeloid-derived suppressor cells (MDSCs), cancer-associated fibroblasts (CAFs), and microglia collectively constitute a major immunoregulatory component of the TME, and accumulating evidence implicates galectins, particularly Gal-1, Gal-3, and Gal-9, as important modulators of this compartment.

Gal-1 drives TAM polarization and Treg recruitment through a convergent signaling architecture across multiple tumor types. In HCC, tumor-secreted Gal-1 activated the PI3K/AKT/NF-κB pathway in TAMs, inducing CCL20 upregulation and the subsequent recruitment of CCR6^+^Foxp3^+^ regulatory T cells; this Treg influx suppressed CD8^+^ T cell infiltration and function, promoting tumor progression and anti-PD-1 resistance, effects reversible by anti-Gal-1 targeting (24). Complementing this, Davuluri et al. identified secretory autophagy as the primary secretion mode for Gal-1 in TAMs: TLR2-dependent autophagosome formation and Rab11/VAMP7-mediated vesicle trafficking released Gal-1 from TAMs into the HCC TME, with this autophagy-regulated secretion correlating with poor overall and progression-free survival in patients (55). Together, these two studies delineate not only the immunosuppressive consequences of TAM-derived Gal-1 but also the intracellular mechanism by which it reaches the extracellular compartment, a distinction relevant to therapeutic targeting. Proteomic analysis of Gal-1-stimulated human macrophages further demonstrated that Gal-1 induces a TAM-like phenotype characterized by upregulated PD-L1 and indoleamine 2,3-dioxygenase-1 (IDO1), with IDO1 induction and kynurenine production occurring in a dose-dependent, JAK/STAT-dependent manner (56). In breast cancer, single-cell RNA sequencing revealed that TAM depletion reduced epithelial-to-mesenchymal transition (EMT) signatures and activated CD8^+^ T cell transcriptional states; Gal-1 was identified as the molecular mediator of these TAM-driven effects, and its inhibition reinvigorated CD8^+^ T cells and potentiated immune checkpoint inhibitors *in vivo* (57).

Gal-1 additionally promotes metastatic progression through myeloid cell manipulation beyond the primary tumor. In head and neck and lung cancer models, tumor-derived Gal-1 established a premetastatic niche by expanding polymorphonuclear MDSCs through NF-κB-driven CXCL2 secretion; mechanistically, Gal-1 stabilized STING protein in tumor cells, sustaining chronic NF-κB activation and systemic MDSC expansion, revealing a context-dependent protumoral role for STING that contrasts with its more commonly described antitumor function (58). In lung cancer, Gal-1 secreted by tumor cells induced TDO2 expression in CAFs via an AKT-dependent pathway, leading to kynurenine production that impaired dendritic cell differentiation and promoted cancer proliferation and EMT; TDO2 inhibition improved dendritic cell function, T cell responses, and reduced metastasis *in vivo* (59). These findings illustrate how Gal-1 orchestrates a multi-cellular immunosuppressive cascade spanning macrophages, MDSCs, and fibroblasts.

Gal-3 exerts complementary myeloid reprogramming through distinct receptors and contexts. In lung cancer, Gal-3 was identified as a ligand for TREM2 on TAMs; Gal-3/TREM2 interaction impaired phagocytosis and promoted conversion of TREM2^+^ macrophages to immunosuppressive TAMs with attenuated antigen presentation, while dual blockade of Gal-3 and TREM2 significantly inhibited tumor progression in subcutaneous and orthotopic models (61). In brain tumors, Gal-3 depletion in microglia and infiltrating macrophages triggered robust interferon-related pro-inflammatory transcriptional remodeling, suppressing cancer cell migration, invasion, and growth in both glioblastoma and breast cancer brain metastasis models (62). Under hypoxia, TAMs upregulate Gal-3 synthesis and secretion via NF-κB/ROS signaling, with this hypoxia-induced Gal-3 promoting tumor growth and metastasis in mammary adenocarcinoma models; combined galectin-3 inhibition and anti-angiogenic therapy produced greater suppression than either agent alone (63). Gal-3 also accelerates M2 macrophage infiltration and tumor angiogenesis: in Gal3^-^/^-^, M2-like macrophages, expressing low Gal-3, preferentially infiltrated tumors and drove enhanced angiogenesis and tumor growth, revealing that host stromal Gal-3 gradient shapes macrophage chemotaxis (64). The exosomal axis linking M2 TAMs to tumor Gal-3 is further illustrated in HCC, where M2 TAM-derived exosomal lncRNA NEAT1 recruited KLF5 to the LGALS3 promoter, upregulating galectin-3 in HCC cells and driving perforin^+^CD8^+^ T cell depletion and immune escape (65). In ovarian cancer, shikonin reduced exosomal Gal-3 delivery to macrophages, blocking β-catenin activation and M2 polarization, thereby restraining tumor growth *in vivo* (66).

Gal-9 extends myeloid reprogramming beyond its canonical T cell-suppressive role. In colon cancer, Gal-9 promoted M2 polarization by binding TIM-3 on macrophages and recruiting PI3K-p85 to the TIM-3 cytoplasmic domain, activating PI3K/Akt signaling and driving arginase-1^+^CD163^+^IL-10^+^ TAM differentiation that increased tumor cell viability, migration, and invasion (81). In metastatic melanoma, plasma Gal-9 was elevated 3.6-fold relative to healthy controls; Gal-9 stimulated monocyte differentiation toward the M2 phenotype *in vitro* and correlated with systemic Th2 polarization and reduced two-year survival in patients (83). At the metastatic frontier, pan-cancer single-cell transcriptomic analysis across 11 cancer types identified a Gal-9^high IL-10-producing macrophages subpopulation specifically enriched in metastatic lymph nodes; Gal-9 activated the BTK/STAT3 pathway in these macrophages, and STAT3 inhibition combined with PD-1 blockade reduced lymph node metastatic burden *in vivo* (90). In muscle-invasive bladder cancer, Gal-9^+^ TAMs predicted poor overall and recurrence-free survival, correlated with Treg and mast cell accumulation, reduced CD8^+^ T and dendritic cell infiltration, and identified patients most likely to benefit from adjuvant platinum-based chemotherapy (82). In cervical cancer, Gal-3 rather than Gal-9 drove MDSC recruitment via STAT3/Akt activation; MDSCs in turn suppressed CD8^+^ T cells through IL-6 and CXCL2, and combined Gal-3/MDSC inhibition reduced subcutaneous tumor growth *in vivo* (67).

**Figure 2:**
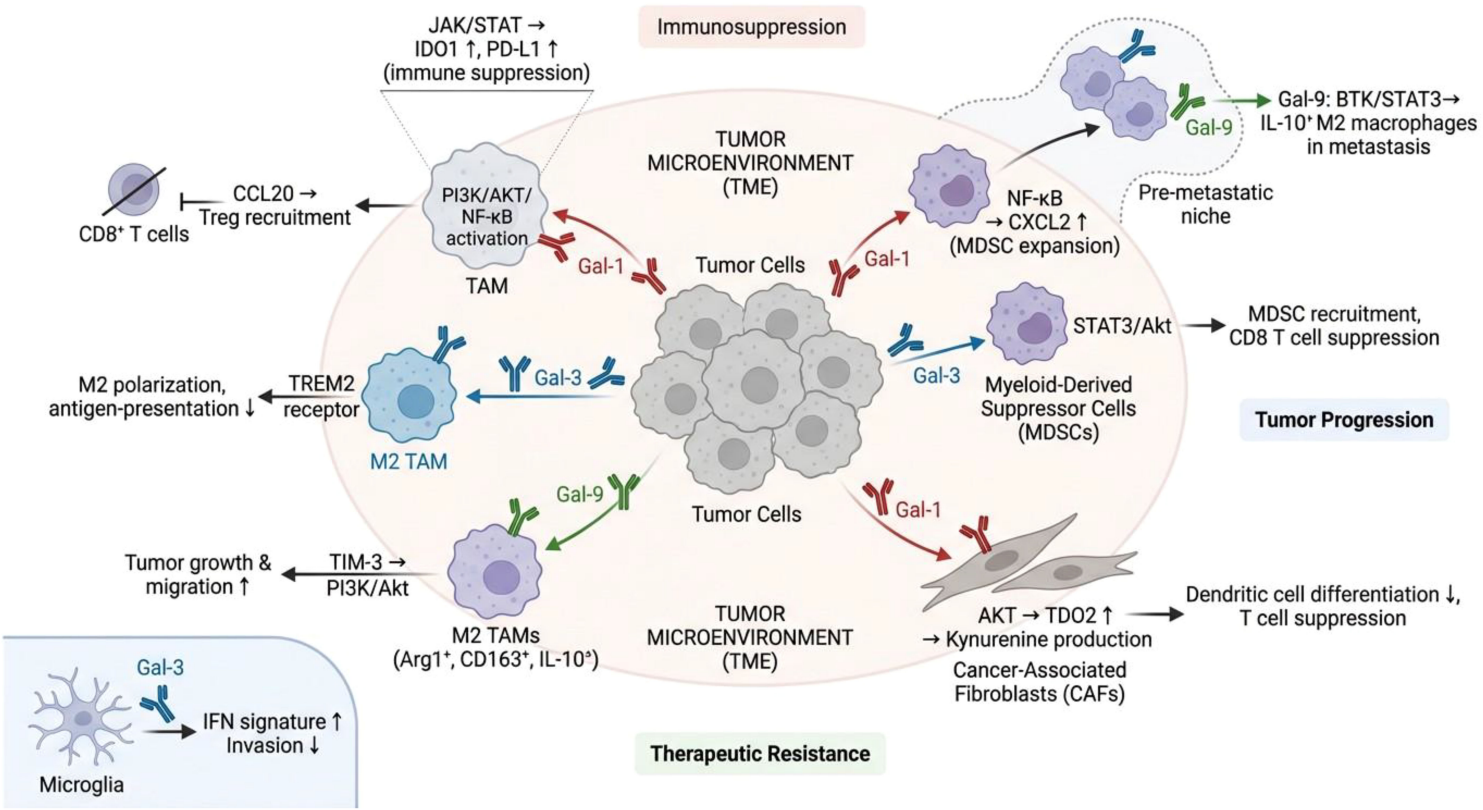
Schematic representation of galectin-mediated reprogramming of myeloid cells within the tumor microenvironment. Gal-1, Gal-3, and Gal-9 promote immunosuppressive remodeling by driving TAM polarization, MDSC expansion, CAF activation, and exosome-mediated intercellular communication through pathways including NF-κB, JAK/STAT, and PI3K/Akt. These myeloid-centered galectin networks suppress anti-tumor immunity, support metastatic niche formation, and highlight galectins as promising targets for combination strategies aimed at restoring immune competence in cancer.

Collectively, these studies indicate that galectins can function as multilevel regulators of the myeloid compartment through macrophage polarization, MDSC expansion, fibroblast activation, and exosome-mediated intercellular communication. A recurring mechanistic theme is the convergence of Gal-1, Gal-3, and Gal-9 signaling on NF-κB, JAK/STAT, and PI3K/Akt-associated pathways within myeloid cells. Myeloid reprogramming by galectins appears to suppress local anti-tumor immunity and, in some settings, may also remodel the systemic and metastatic immune landscape, an important consideration for therapeutic strategies aimed at restoring immunological competence in advanced-stage disease (**Figure 2**).

### 3.3 Galectin regulation of innate effector cells: NK cells, neutrophils, and dendritic cells

Whereas sections 4.1 and 4.2 highlighted prominent roles for galectins in adaptive immune suppression and myeloid cell reprogramming, their influence also extends to the innate effector compartment. Natural killer (NK) cells, neutrophils, and dendritic cells (DCs) are each subject to galectin-mediated regulation, and the functional consequences range from direct cytotoxic suppression to paradoxical activation, reflecting the context dependence characteristic of this protein family.

In glioma, Gal-1 suppresses an endogenous innate anti-tumor circuit. Tumor-derived exosomes carry miR-1983, which activates TLR7 in plasmacytoid and conventional DCs via a 5’-UGUUU-3’ motif, triggering MyD88-IRF5/IRF7 signaling and IFN-β secretion; IFN-β then licenses NK cells to kill glioma cells. Gal-1 negatively regulates this miR-1983/TLR7/IFN-β axis, thereby preventing NK cell activation and enabling immune escape (60). These observations suggest that, in this model, Gal-1 may restrain both innate and adaptive antitumor responses through a shared regulatory node. Contrasting with this suppressive role, Gal-9 can potentiate innate killing. Treatment of mixed neutrophil-tumor cell cultures with Gal-9 rapidly externalized phosphatidylserine on cancer cells, downregulated the anti-phagocytic signal CD47, and activated neutrophils with mobilization of gelatinase and secretory granules, resulting in augmented trogocytosis and antibody-dependent cellular phagocytosis of cancer cells; neutrophil depletion abrogated this cytotoxicity (85). This pro-phagocytic effect of Gal-9 stands in marked contrast to its T cell-suppressive function described in section 4.1, underscoring the cell-type specificity of galectin signaling.

**Figure 3:**
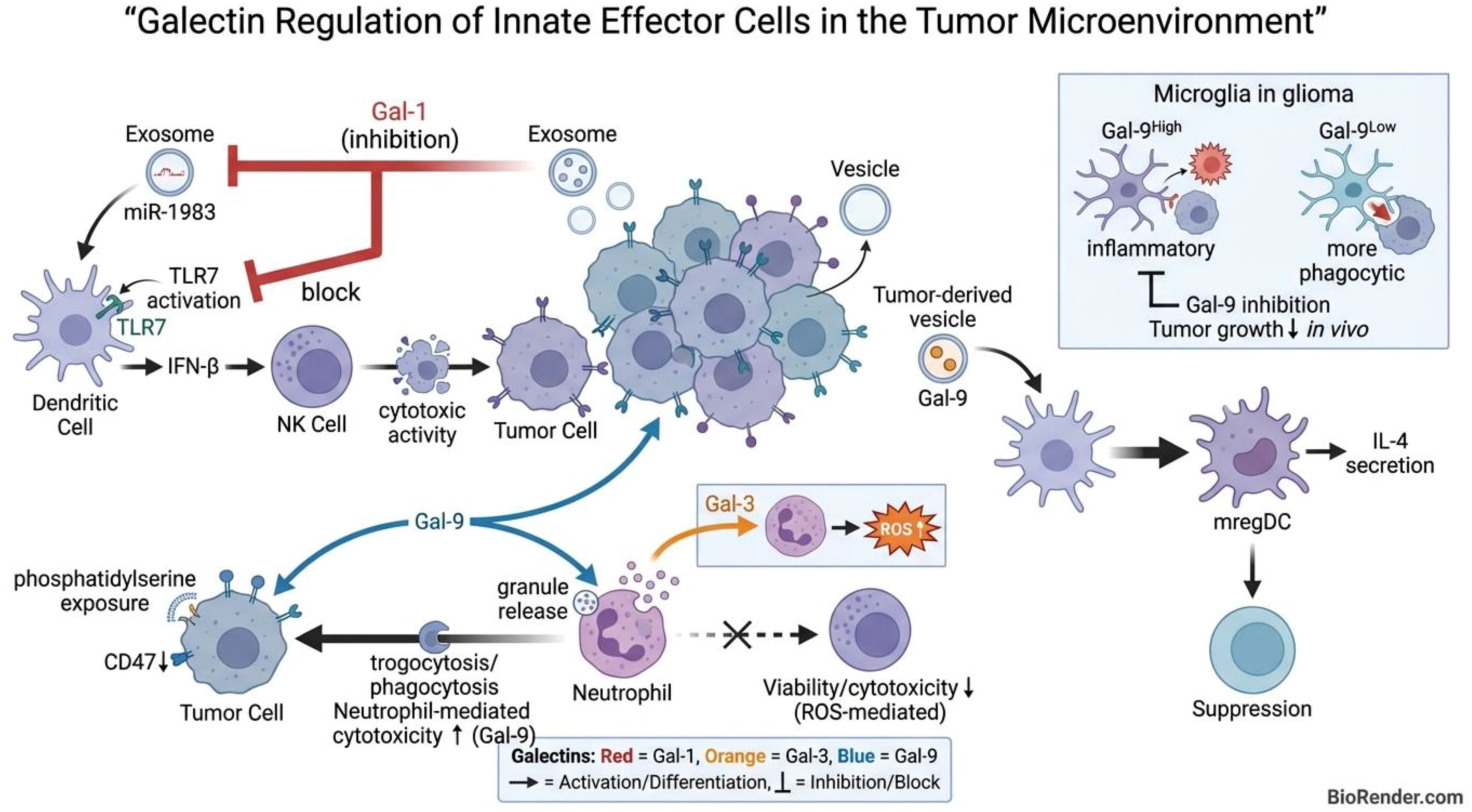
Schematic overview of galectin-mediated regulation of innate effector cells in the tumor microenvironment. Gal-1, Gal-3, and Gal-9 modulate NK cells, neutrophils, dendritic cells, and microglia through distinct mechanisms that can either suppress or enhance innate anti-tumor responses, depending on cellular context. These findings highlight the dual and context-dependent roles of galectins in controlling cytotoxicity, phagocytosis, and tolerogenic reprogramming, supporting their relevance as nuanced targets in cancer immunotherapy.

Gal-3 introduces an additional immunosuppressive node through neutrophils. In high-grade serous ovarian carcinoma, Gal-3 was elevated in both ascites and cyst fluid; it induced reactive oxygen species (ROS) production in tumor-associated neutrophils, which in turn impaired NK cell viability and cytotoxicity in co-culture assays, though the effect was heterogeneous and correlated with neutrophil priming status across patients (68). In nasopharyngeal carcinoma, tumor-derived extracellular vesicles carrying Gal-9 converted DCs into mature regulatory DCs (mregDCs) with IL-4-secreting, T cell-suppressive phenotypes; anti-Gal-9 neutralization reversed this tolerogenic conversion, implicating vesicular Gal-9 as a key mediator of DC dysfunction (84). Finally, in glioma, microglial Gal-9 expression is regulated by hypoxia-inducible factor-2α and defines functionally distinct subsets: Gal-9^high microglia display inflammatory activation but reduced phagocytic capacity, whereas Gal-9^low microglia phagocytose tumor cells more effectively; Gal-9 blockade suppressed tumor growth *in vivo* (86, 87). Together, these findings highlight the innate immune compartment as an important, and in some settings under-recognized, context of galectin activity with distinct and sometimes opposing therapeutic implications (**Figure 3**).

## 4 Metabolic-immune crosstalk: galectins as metabolic rheostats

Moving beyond immune regulation, this section examines how galectins function as metabolic rheostats within the tumor ecosystem. We discuss their roles in glycolytic reprogramming and glycan remodeling, their emerging involvement in ferroptosis regulation and the functional divergence between Gal-13 and Gal-1, and their influence on lipid metabolism, mitochondrial function, and immunometabolic reprogramming, collectively revealing a metabolic dimension of galectin biology with direct implications for therapeutic resistance (**Table 2**).

**Table 2:** Galectin-mediated regulation of cellular metabolism, ferroptosis resistance, and drug response in cancer and non-cancer contexts.

Galectin	Cancer/disease	Study design	Metabolic pathway	Key findings	Ref.
	HCC	TCGA; AAV gene therapy; Spatial transcriptomics; Orthotopic model	Glycolysis/O-glycosylation/Immunity	miR-22 suppresses Gal-1; miR-22-high/Gal-1-low = best HCC survival. Anti-HCC effect of miR-22 is Gal-1-dependent. Gal-1 silencing (siRNA/LLS30) mimics miR-22 anti-tumor effects.	(91)
	HCC	*In vitro* (Huh-7/SR sorafenib-resistant line); *In vivo*	Ferroptosis resistance/MET–AXL signaling	Gal-1 elevated in sorafenib-resistant HCC; KD restores sensitivity. Gal-1 inhibits lipid peroxidation → escapes sorafenib-induced ferroptosis. Gal-1 activates AXL and MET phosphorylation; AXL correlated with Gal-1.	(92)
	Gastric Cancer (Peritoneal metastasis)	Clinical IHC; Lentiviral model; Transcriptomics; Molecular docking	Ferroptosis resistance/PI3K–Akt–Nrf2–HO-1	Gal-1 activates PI3K/Akt/Nrf2/HO-1 → ferroptosis resistance → peritoneal spread. DHA targets this pathway; blocks Gal-1-induced metastasis *in vitro* & *in vivo*. Molecular docking confirms DHA directly inhibits Gal-1 signaling.	(93)
	Acute Myeloid Leukemia	TCGA bioinformatics (Cox); *In vitro*; *In vivo*	Fatty acid/lipid metabolism (LSC stemness)	LGALS1 is the key gene of a fatty acid risk score (LFMRS) for AML prognosis. LGALS1 KD inhibits LSC proliferation and ↓lipid accumulation *in vitro*. *In vivo*: LGALS1 KD curbs AML and ↑CD8+ T and NK cell counts.	(94)
	Atherosclerosis (Non-cancer)	*In vivo* (Apoe−/− mice); Ex vivo foam cell; Human endarterectomy	Macrophage immunometabolic reprogramming	Gal-1 reduces plaque size; shifts macrophages to anti-inflammatory/quiescent state. ↑IL-10, IL-17, IL-22, IL-23 and Tregs via glycan-dependent & -independent pathways. Protective effect partly IL-10-independent (Gal-1E71Q variant).	(95)
Galectin-3	Pancreatic Adenocarcinoma	Clinical; Co-culture (PAAD + stellate cells); Orthotopic xenograft	Mitochondrial OXPHOS suppression/Gemcitabine resistance	Stromal Gal-3 activates Ca^2+^/calcineurin-NFAT → ↑CCL2 and BSG in fibroblasts. Gal-3-high tumor cells: arrested OXPHOS (↓OCR, ↓ATP, abnormal mitochondria). CCL2-CCR2/PPIA-BSG blockade or oral MCP restores gemcitabine sensitivity.	(21)
Galectin-4	Colorectal Cancer	TCGA + GEO; *In vitro* (CRC lines); LASSO-Cox	Aerobic glycolysis/β-catenin	LGALS4 downregulated ~3-fold in CRC; low expression → poor survival. LGALS4 OE inhibits glycolysis; effects amplified by XAV-939 (β-catenin inhibitor). LGALS4 OE: ↓proliferation ~50%, ↑5-FU apoptosis ~2-fold, cell cycle arrest.	(96)
	Gastric Cancer (Peritoneal metastasis)	*In vitro* (NUGC4 WT/KO/rescue); MS + glycosidase digestion	Glycosphingolipid glycan remodeling	Gal-4 KO alters neutral GSL profile in high-metastatic GC cells (not N-glycans). Shift from neolacto (β1-4Gal) to lacto-type (β1-3Gal) GSL structure by MS. Gal-4 regulates GSL glycosylation and thereby modulates metastatic potential.	(97)
Galectin-9	Gastric Cancer (Liver/LN metastasis)	scRNA-seq (primary + metastases); *In vitro*; P4HB inhibition	Lipid/cholesterol metabolism/PPAR/EMT	Myeloid LGALS9 binds P4HB on epithelial cells — novel ligand-receptor pair. LGALS9-P4HB ↑proliferation, EMT, and lipid/cholesterol metabolism at metastatic sites. P4HB inhibition reverses LGALS9-driven effects; proposed as therapeutic target.	(98)
Galectin-13	Multiple cancers (Pan-cancer)	*In vitro*; *In vivo*; Synthetic mimetic peptide	Ferroptosis propagation/SLC7A11 membrane trafficking	Ferroptotic cells secrete Gal-13 → binds CD44 → ↓SLC7A11 on membrane → propagates ferroptosis. PKCβII phosphorylates FOXK1 → drives Gal-13 secretion during ferroptotic death. Gal-13 mimetic peptide sensitizes tumors to erastin, RT & immunotherapy; CSCs vulnerable.	(99)
	Pre-eclampsia (Structural biology)	X-ray crystallography; Mutagenesis (C136S, C138S); HeLa cells	Redox regulation/Disulfide dimerization	Gal-13 dimerizes via Cys136–Cys138; all mutants lose hemagglutination activity. C136S forms alternative Cys138–Cys19 bond; dimerization alone insufficient for function. DelT221 variant cannot enter nucleus → likely pathogenic mechanism in pre-eclampsia.	(100)

### 4.1 Galectin regulation of tumor cell metabolism and glycosylation

Beyond immune modulation, galectins regulate intrinsic tumor cell biology, including metabolic reprogramming and glycan remodeling, thereby shaping malignant progression through mechanisms that are independent of immune evasion yet intersect with therapeutic resistance. In CRC, LGALS4 functions as a metabolic tumor suppressor. Its expression is reduced approximately threefold relative to normal colonic tissue and correlates with adverse patient survival. LGALS4 overexpression inhibited aerobic glycolysis and glucose-dependent metabolic activity, suppressed cell proliferation by approximately 50%, enhanced apoptosis twofold, and sensitized CRC cells to 5-fluorouracil. These effects were amplified by co-treatment with the β-catenin inhibitor XAV-939, positioning LGALS4 as a regulator of both Warburg metabolism and Wnt/β-catenin signaling (96).

In HCC, Gal-1 appears to exert an opposing effect by promoting metabolic flexibility in association with treatment resistance. Proteomic profiling of thermal ablation specimens identified Gal-1 overexpression in non-responders compared with responders. Mechanistically, Gal-1 facilitated GM1-ganglioside breakdown to generate galactose, increasing flux into glycolysis and the downstream TCA cycle under hyperthermia-induced stress. Combined Gal-1 inhibition and thermal ablation reduced tumor size significantly beyond either monotherapy *in vivo*, supporting the view that Gal-1 contributes to metabolic resilience in HCC (101). Complementing this, analysis of the miR-22/Gal-1 axis in HCC revealed that miR-22-high/Gal-1-low tumors have the best survival outcomes. miR-22 gene therapy and Gal-1 silencing converged on shared intracellular signaling pathways, and the anti-HCC efficacy of miR-22 was dependent on Gal-1 suppression; both interventions also reduced O-linked glycosylation, implicating the axis in modifying extracellular Gal-1 function (91).

**Figure 4:**
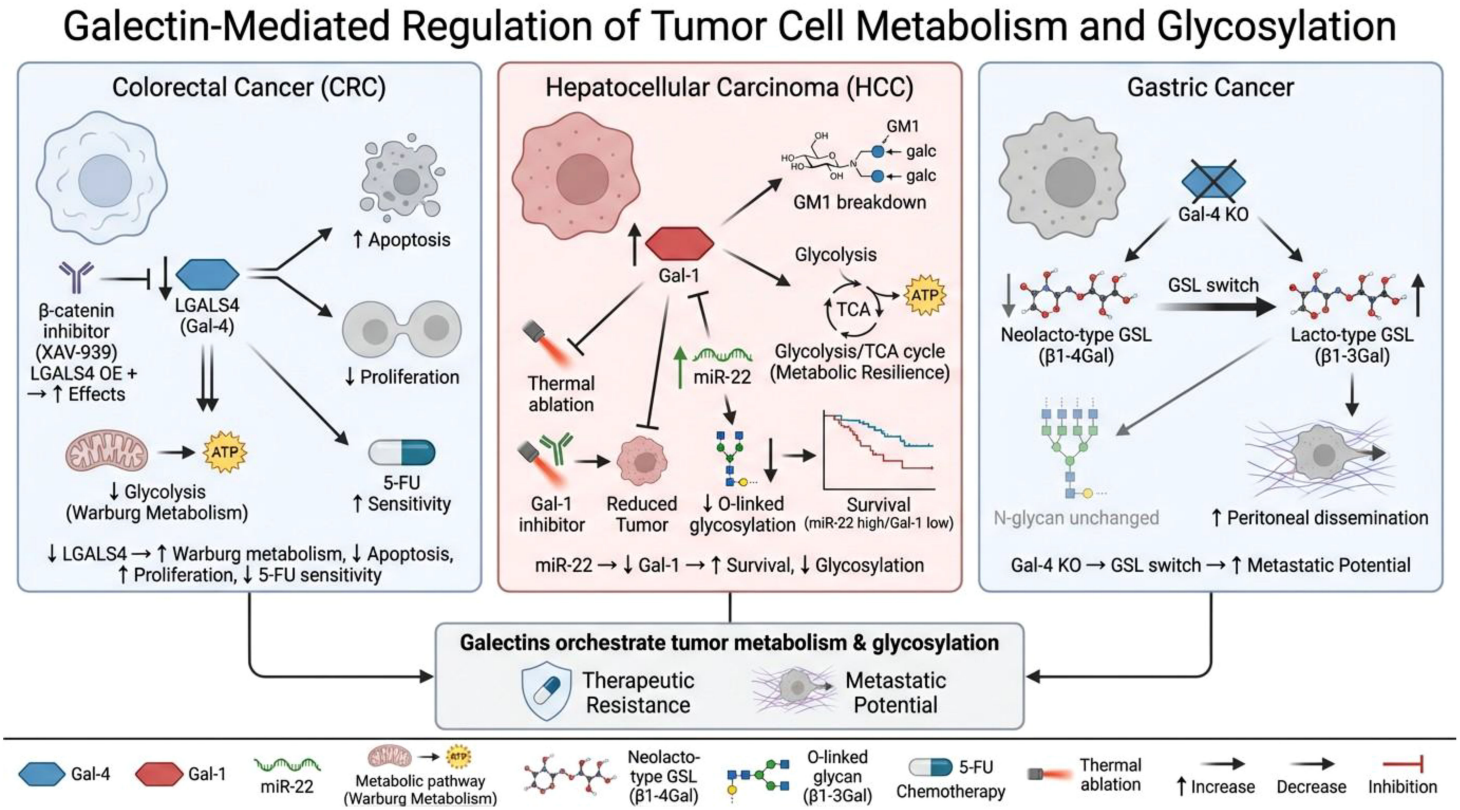
Schematic illustration of galectin-mediated regulation of tumor cell metabolism and glycosylation. Galectins differentially shape intrinsic tumor cell behavior, with Gal-1 promoting metabolic flexibility and treatment resistance, LGALS4 acting as a suppressor of aerobic glycolysis and tumor growth, and Gal-4 remodeling glycosphingolipid glycan structures linked to metastatic potential. These findings position galectins as important regulators at the interface of cancer metabolism and glycobiology, with implications for therapeutic sensitivity, malignant progression, and metastasis.

Gal-4 contributes a distinct dimension by remodeling the glycan landscape of tumor cell surfaces. In poorly differentiated gastric cancer cells with high peritoneal dissemination potential, Gal-4 knockout induced marked structural changes in neutral glycosphingolipid (GSL) glycans, specifically shifting from neolacto-type (β1-4Galactosyl) to lacto-type (β1-3Galactosyl) GSL, without altering N-glycan profiles, identifying Gal-4 as a candidate regulator of GSL glycosylation that may facilitate peritoneal metastasis (97). Together, these studies indicate that galectins participate at the interface of tumor metabolism and glycobiology, with implications that may extend beyond immunity to therapeutic resistance and metastatic capacity (**Figure 4**).

### 4.2 Galectins at the interface of ferroptosis and therapeutic resistance

Complementing their roles in metabolic reprogramming described in section 5.1, galectins have recently emerged as regulators of ferroptosis, an iron-dependent, lipid peroxidation-driven cell death pathway of increasing relevance to cancer therapy. The functional roles identified to date are divergent: certain galectins promote ferroptosis propagation, while others confer ferroptosis resistance, with direct consequences for drug sensitivity and metastatic behavior. Current evidence suggests that Gal-13 can function as a paracrine amplifier of ferroptosis. Cells undergoing ferroptosis secrete Gal-13, which binds CD44 on neighboring cells and inhibits plasma membrane localization of the cystine transporter SLC7A11, thereby reducing antioxidant defense and propagating cell death to adjacent cells. PKCβII-mediated phosphorylation of FOXK1 drives Gal-13 expression and secretion during ferroptotic death. In preclinical models, a synthetic Gal-13 mimetic peptide sensitized tumors, including cancer stem cells, to the ferroptosis inducer imidazole ketone erastin, radiotherapy, and immunotherapy, supporting the idea that ferroptosis propagation capacity may influence overall ferroptosis sensitivity (99). Structural studies further clarified that Gal-13 dimerization and function are controlled by redox-active disulfide bonds between Cys136 and Cys138; mutation of these residues abolished hemagglutination activity and altered cellular distribution, indicating that the redox environment governs Gal-13 conformation and bioactivity (100).

Gal-1, by contrast, promotes ferroptosis resistance. In sorafenib-resistant HCC cells, Gal-1 expression was markedly elevated, and its knockdown restored sorafenib sensitivity. Mechanistically, Gal-1 inhibited lipid peroxidation and activated AXL and MET receptor tyrosine kinase signaling to protect cells from sorafenib-mediated ferroptosis (92). In gastric cancer, Gal-1 activated the PI3K/Akt/Nrf2/HO-1 pathway to suppress ferroptosis, facilitating peritoneal metastasis; dihydroartemisinin (DHA) targeted this pathway, inhibited Gal-1-induced ferroptosis resistance, and reduced peritoneal dissemination *in vivo* (93). Collectively, the Gal-13/Gal-1 contrast illustrates a functional dichotomy within the galectin family with respect to ferroptosis regulation and suggests that ferroptosis modulation may represent a therapeutically relevant strategy for counteracting galectin-associated treatment resistance.

### 4.3 Galectins in lipid metabolism, mitochondrial function, and immunometabolic reprogramming

Extending the metabolic themes established in sections 5.1 and 5.2, galectins also regulate lipid metabolism and mitochondrial energy homeostasis, processes central to tumor stemness, drug resistance, and, beyond oncology, to immunometabolic diseases such as atherosclerosis. In acute myeloid leukemia (AML), LGALS1 was identified as the central gene of a fatty acid metabolism-related risk score model derived from bioinformatic integration of TCGA and GEO datasets. LGALS1 was highly expressed in leukemia stem cells (LSCs) and correlated with poor prognosis. Its repression inhibited AML cell and LSC proliferation, enhanced apoptosis, and reduced intracellular lipid accumulation *in vitro*; *in vivo*, LGALS1 knockdown curbed AML progression alongside reductions in lipid burden and CD8^+^ T and NK cell infiltration, linking Gal-1-driven lipid accumulation to both stemness maintenance and immune suppression (94). In metastatic gastric cancer, single-cell transcriptomic analysis identified myeloid-derived Gal-9 (LGALS9) and its epithelial receptor P4HB as a previously uncharacterized ligand-receptor pair upregulated in liver and lymph node metastases relative to primary tumors; P4HB activation by LGALS9 enhanced cholesterol metabolism, PPAR signaling, EMT, and cancer cell proliferation, while pharmacological P4HB inhibition reversed these effects, implicating the LGALS9-P4HB axis in lipid-driven metastatic colonization (98).

At the mitochondrial level, stromal Gal-3 in pancreatic adenocarcinoma activated the Ca^2+^/calcineurin-NFAT pathway in tumor-associated fibroblasts, upregulating CCL2 and BSG, which suppressed mitochondrial oxidative phosphorylation in tumor cells, evidenced by reduced oxygen consumption and ATP production and abnormal mitochondrial morphology. This Gal-3-mediated energy metabolic arrest conferred gemcitabine resistance, which was reversed by inhibiting the CCL2-CCR2 and PPIA-BSG axes or by oral administration of the natural Gal-3 inhibitor modified citrus pectin in orthotopic xenograft models (21). Beyond cancer, Gal-1 reprogrammed macrophage immunometabolism in a murine atherosclerosis model, promoting anti-inflammatory, metabolically quiescent foamy macrophage phenotypes and reshaping T cell cytokine profiles through glycan-dependent and -independent mechanisms, reducing plaque burden (95). This non-oncological context underscores the broader relevance of galectin-driven metabolic reprogramming across immunometabolic disease states.

## 5 EMT, invasion, and metastatic niche formation

Building on the immune and metabolic roles described in preceding sections, this section addresses how galectins drive tumor dissemination across the metastatic cascade. We trace their involvement from epithelial–mesenchymal transition and local invasion, through vascular adhesion, platelet crosstalk, and hematogenous dissemination, to stromal remodeling, peritoneal niche formation, and cancer stem cell-mediated colonization at distant sites.

### 5.1 Galectins as drivers of epithelial–mesenchymal transition and tumor invasion

Having outlined the roles of galectins in tumor metabolism and cell death in section 5, evidence also links several family members to the regulation of tumor plasticity and invasiveness. EMT represents a convergence point at which galectin-associated signaling may facilitate local invasion and distant metastasis. Gal-1 is among the most consistently implicated galectins in EMT induction across tumor types. In HCC, forced Gal-1 expression elevated αvβ3-integrin, activated the FAK/PI3K/AKT cascade, and induced EMT; Gal-1 overexpression also correlated clinically with poor survival and reduced sorafenib response, supporting its role as a driver of invasive behavior and a candidate predictive biomarker in this setting (102). In gastric cancer, CAF-derived Gal-1 operated through two convergent paracrine mechanisms: β1-integrin binding activated hedgehog signaling and upregulated Gli1 (103), while TGF-β1/Smad pathway activation drove EMT and *in vivo* metastasis, effects reversed by TGF-β1 antagonism (104). The identification of distinct downstream pathways from the same Gal-1/β1-integrin interaction in two independent gastric cancer studies suggests that this interaction can engage more than one signaling route. In breast cancer, the consequences of Gal-1 are subtype-dependent: knockdown in hormone receptor-positive cells induced G0/G1 cell cycle arrest, whereas in triple-negative cells it suppressed EMT markers; miR-22-3p, an endogenous Gal-1 suppressor, inversely correlated with Gal-1 and was associated with improved metastasis-free survival (105). This subtype specificity suggests that galectin-targeting strategies may require contextualization within tumor molecular classification.

Gal-3 contributes independently, though with context-dependent directionality. In lung adenocarcinoma, cigarette smoke activated the ROS/JNK-MAPK axis to co-upregulate RUNX2 and Gal-3, driving EMT, spheroid formation, and pro-inflammatory cytokine secretion; pharmacological JNK inhibition reversed all these effects, identifying the ROS/JNK/RUNX2/Gal-3 axis as a targetable driver of carcinogen-induced oncogenic plasticity (106). In urothelial carcinoma, Gal-3 was highest in T1 high-grade tumors yet decreased in T2 stage tumors; despite classical EMT markers correlating with grade, no direct correlation between Gal-3 and E-cadherin or vimentin was found (107), suggesting Gal-3 may be more relevant to early invasion than to advanced metastatic EMT. Collectively, the available evidence supports the view that galectins, particularly Gal-1 and Gal-3, function as context-dependent regulators of tumor invasion through integrin, TGF-β, hedgehog, and MAPK-associated pathways. The recurring involvement of miRNA-mediated suppression (miR-22-3p) connects invasion biology to the transcriptional regulatory mechanisms explored in earlier sections and supports the rationale for combined galectin inhibition and endogenous suppressor restoration as a therapeutic strategy.

### 5.2 Galectins in vascular adhesion, platelet crosstalk, and metastatic dissemination

Whereas section 6.1 addressed galectin-driven invasion at the primary tumor site, successful metastatic dissemination requires additional steps: intravasation, survival in the circulation, endothelial adhesion, and extravasation. Galectins contribute mechanistically to each of these stages, operating at the interface between circulating tumor cells, the vascular endothelium, and platelets.

A foundational observation is that multiple galectin family members are elevated in patient serum, with galectin-2, -3, -4, and -8 increased up to 31-fold in colon and breast cancer patients, particularly those with metastases. Each promoted cancer cell adhesion to microvascular endothelium by interacting with the Thomsen-Friedenreich (TF) disaccharide on cancer-associated MUC1, inducing MUC1 polarization and exposing underlying adhesion molecules; elevated serum galectin-2 further correlated with increased colorectal cancer mortality, attenuated when anti-MUC1/TF antibodies were co-present (108). Gal-3 amplifies this adhesion through an extracellular vesicle-mediated route: circulating Gal-3 upregulated glycolysis in vascular endothelial cells, increasing ICAM-1 expression; ICAM-1 was packaged into endothelium-derived vesicles and transferred to triple-negative breast cancer cells, enhancing tumor-endothelium adhesion, reversible by targeting either cirGal-3 or endothelial glycolysis (109).

At the intravascular level, tumor cell-surface Gal-3 engages platelet glycoprotein VI (GPVI), a collagen/fibrin receptor transducing ITAM signaling via Syk. Genetic deficiency of platelet GPVI or Syk reduced both experimental and spontaneous colon and breast cancer metastasis in mice; CRISPR screens confirmed tumor Gal-3 as the principal GPVI counter-receptor, and GPVI inhibition impaired platelet-tumor cell interaction and extravasation *in vivo* (110), positioning Gal-3/GPVI crosstalk as a tractable anti-metastatic target. In cervical cancer, the scaffold protein RACK1 promoted lymphangiogenesis and lymph node metastasis in a Gal-1-dependent manner: RACK1 suppressed miR-1275 to increase Gal-1 secretion, which activated MEK/ERK, FAK, and AKT via β1-integrin (111). In bladder urothelial carcinoma, Gal-1 drove invasion through the Ras-Rac1-MEKK4-JNK-AP1 axis to upregulate MMP9; Gal-1 silencing reduced proliferation, invasion, and clonogenicity (112). In HCC, Gal-3 promoted hepatoma cell migration via RhoA GTPase activation and MLC2 phosphorylation; Gal-3-deficient mice developed significantly smaller and less invasive tumors (113).

In breast cancer, Gal-9 promoted extracellular matrix invasion by inducing FAK activity and upregulating S100A4; pharmacological Gal-9 inhibition reduced cancer cell adhesion to and invasion through organomimetic matrix microenvironments (114). In gastric adenocarcinoma, loss of the E3 ubiquitin ligase TRIM49 stabilized Gal-3 by preventing polyubiquitination and proteasomal degradation; accumulated Gal-3 assembled a transcriptional complex with EGR1 to activate a pro-invasive gene module, and the oral Gal-3 inhibitor GB1107 suppressed tissue infiltration and metastasis of patient-derived xenografts (115). Compared with the family members discussed above, Gal-8 has received comparatively little attention in the tumor context, yet emerging evidence identifies it as an additional regulator of the immune microenvironment and of metastasis (116). In murine models, Gal-8 induced the expression and secretion of cytokines and chemokines, including SDF-1 and MCP-1, through a uPAR/LRP1/integrin complex that activates JNK and NF-κB signaling; Gal-8-knockout mice displayed lower systemic cytokine levels and developed smaller primary tumors with fewer metastatic lesions, suggesting that tumor-derived Gal-8 sustains a self-reinforcing chemoattractant loop at the metastatic niche (117). Consistent with an immunoregulatory role, Gal-8 expression in tumors was associated with expansion of regulatory T cells and myeloid-derived suppressor cells, a concomitant reduction in CD8^+^ T cells, and correlated with lymph node metastasis across breast and colorectal cancer datasets (118). Given these activities, Gal-8 has been proposed as a prognostic indicator and a candidate therapeutic target, and the development of selective Gal-8 inhibitors is now under active investigation (116). Collectively, these findings reveal that galectins orchestrate metastatic dissemination through a coordinated network spanning vascular adhesion, hematological crosstalk, cytoskeletal remodeling, and transcriptional reprogramming, several of which may represent potential therapeutic targets.

### 5.3 Galectins in tumor-stroma crosstalk, peritoneal metastasis, and cancer stem cell biology

Complementing vascular dissemination mechanisms described in section 6.2, galectins also promote metastasis by remodeling stromal compartments and creating permissive niches at distant sites. Peritoneal dissemination, a particularly lethal route in gastrointestinal cancers, and early lymph node colonization by cancer stem cells represent two contexts in which this dimension of galectin biology is especially consequential. In pancreatic ductal adenocarcinoma (PDA), stromal Gal-1 appears to function as an important regulator of disease progression. Genetic deletion of Gal-1 in a Kras-driven mouse model extended survival through simultaneous suppression of stromal activation, angiogenesis, and metastasis, alongside enhanced T cell infiltration; orthotopic co-injection of tumor cells with Gal-1-depleted human pancreatic stellate cells impaired tumor formation *in vivo* (119). Extracellular Gal-4, independently identified in PDAC tumors and patient circulation, deposited into the extracellular matrix and induced T cell apoptosis by binding N-glycosylation residues on CD3ϵ/δ; Gal-4 knockdown increased T cell and M1 macrophage infiltration, shifted CAF composition toward myofibroblastic subtypes, and extended survival in immunocompetent but not RAG1^-^/^-^ mice, confirming adaptive immune dependency (120). These two studies in PDAC identify Gal-1 and Gal-4 as stromal and matrix-deposited drivers of immune exclusion, respectively.

In gastric cancer, Gal-1 promotes peritoneal metastasis through two complementary mechanisms: induction of peritoneal fibrosis via collagen I, collagen III, and fibronectin-1 upregulation in mesothelial cells, correlated with clinical staging in patient tissue (121), and engagement of the chaperone GRP78 in tumor cells to promote proliferation and invasion; GRP78 knockdown abolished Gal-1 overexpression effects (122). Exosomal Gal-3 extends peritoneal remodeling to the ascitic compartment: identified as the dominant cargo in malignant ascites exosomes, it activated CAFs via integrin α1β1/FAK/Akt/mTOR/CXCL12 signaling; combined CXCL12-CXCR4 inhibition and exosomal Gal-3 blockade synergized with anti-PD-1 therapy (123). Gal-4 also contributes in poorly differentiated gastric cancer: its knockout reduced c-MET and CD44 activation, suppressed proliferation, and attenuated peritoneal dissemination *in vivo*; Gal-4 expression was confirmed in peritoneal metastatic tumor cells across all patients examined (124). LGALS9B, a paralogue of galectin-9 not previously characterized in this context, stabilized the translation elongation factor EEF1D by competing with E3 ligase HERC5, thereby activating PI3K/AKT signaling and promoting gastric cancer proliferation, migration, and metastasis (125).

In prostate cancer, Gal-3 was overexpressed specifically in cancer stem-like cells (CSCs) colonizing draining lymph nodes; Gal-3 silencing or pharmacological inhibition with N-acetyl-D-lactosamine rescued T cell proliferation and reduced both primary tumor growth and secondary invasion, while nuclear/cytoplasmic Gal-3 distribution in CSCs conferred relative apoptosis resistance (8). Collectively, these findings suggest that galectins function as multi-modal regulators of the tumor-stroma interface, peritoneal niche formation, and stem cell-mediated early dissemination.

## 6 Galectins in therapeutic resistance

The diverse biological functions of galectins documented in preceding sections—spanning immune evasion, metabolic adaptation, and metastatic progression—converge on a clinically consequential outcome: resistance to therapy. This section examines how galectins contribute to resistance against cytotoxic chemotherapy, targeted agents, and hormonal therapies, and subsequently addresses their emerging roles in resistance to immune checkpoint inhibitors and CAR-T cell-based approaches (**Table 3**).

**Table 3:** Galectin-mediated resistance to chemotherapy, targeted therapy, and immunotherapy across cancer types.

Galectin	Cancer type	Drug/therapy	Resistance mechanism	Key finding	Ref.
Chemotherapy & targeted therapy resistance					
Galectin-1	HCC	Doxorubicin (DOX)	Gal-1 → PI3K → ↑P-gp expression → ↓intracellular DOX	Gal-1 OE → DOX-resistant tumors *in vivo*. P-gp mediates Gal-1-driven DOX (not sorafenib) resistance.	(126)
	Prostate Cancer	Enzalutamide	Gal-1 → ↑AR/AR-V7 expression and transcriptional activity	Gal-1 KD resensitizes resistant cells. LLS30 + enzalutamide synergistically inhibits resistant xenografts.	(127)
	Esophageal SCC	Paclitaxel	Gal-1 → Wnt/β-catenin → ↑MDR1 transcription and cancer stemness	Serum Gal-1 elevated in resistant patients. OTX008 + paclitaxel reverses resistance *in vitro* & *in vivo*.	(128)
	Gastric Cancer	Cisplatin	CAF-secreted Gal-1 → NRP-1/c-JUN/Wee1 pathway activation	Gal-1 promotes proliferation, metastasis, and cisplatin resistance. NRP-1 inhibitor EG00229 counteracts effects.	(129)
	Ovarian Cancer	Cisplatin/Paclitaxel	SNHG22 sponges miR-2467 → releases Gal-1 mRNA → PI3K/AKT + ERK activation	SNHG22 OE drives chemo-resistance; SNHG22 KD restores cisplatin/paclitaxel sensitivity in EOC.	(130)
Galectin-1 (protective)	Lung Cancer (CAF model)	Anlotinib	Gal-1 OE converts normal fibroblasts → CAFs; CAF-CM ↑cancer cell motility, ↑Bcl-2, ↓apoptosis	CAF-derived Gal-1 reduces anlotinib sensitivity; Gal-1 OE promotes tumor growth and angiogenesis *in vivo*.	(131)
Galectin-3	HER2+ Breast Cancer	Trastuzumab	Gal-3 → PI3K/AKT (malignancy) + Notch1 (stemness)	LGALS3 KO synergizes with trastuzumab *in vitro* & *in vivo*. High LGALS3 = poor prognosis.	(132)
	Colorectal Cancer	5-Fluorouracil	BAP31 → Gal-3 → Wnt/β-catenin (c-MYC, SOX2) → ↑stemness	BAP31 KD ↓Gal-3 → ↓β-catenin → restores 5-FU sensitivity and suppresses stemness *in vivo*.	(133)
	Liver (non-cancer) Cisplatin toxicity	Cisplatin (hepatotoxicity model)	Endogenous Gal-3 in hepatocytes is hepatoprotective; Gal-3 inhibition (MCP) ↑oxidative stress and cell death	Gal-3 protects liver from cisplatin-induced ROS and inflammation. Gal-3 reduction ↑MDA, ↑IL-1β, ↑TNF-α.	(134)
	Ovarian Cancer	Cisplatin	DMBT1 binds Gal-3 → ↓Gal-3 → inhibits PI3K/AKT phosphorylation	DMBT1 OE reduces proliferation/invasion and sensitizes SKOV3 cells to cisplatin.	(135)
	Cervical Cancer (sensitizer role)	Cisplatin	Gal-7 → RACK1/G3BP1 axis → stress granule clearance → ↑ROS, ↑apoptosis	Gal-7 promotes CDDP-induced apoptosis; reduces chemoresistance by clearing stress granules.	(136)
Galectin-9	AML	Cytarabine (AraC)	Gal-9 induces non-apoptotic, autophagy-linked cytotoxicity independent of AraC pathway	Gal-9 kills AraC-sensitive and -resistant AML cells and CSCs; potentiates Azacytidine in AraC-ineligible patients.	(137)
Immunotherapy & CAR-T resistance					
Galectin-1	NSCLC	Anti-PD-1 (nivolumab/pembrolizumab)	Gal-1 in plasma & tumor tissue dampens T-cell cytotoxicity against tumor cells	High Gal-1 → trend toward shorter PFS. Gal-1 KO tumor cells more susceptible to T-cell killing (BiTE assay).	(138)
	Renal Cell Carcinoma	IO + TKI combination	High Gal-1 → ↓GZMB+CD8+ T cells, ↑PD-1+CD8+ T cells, ↑Tregs, ↑CAFs	Gal-1 independently predicts shorter PFS (HR 2.51). IO/TKI benefit restricted to low-Gal-1 patients.	(139)
	Multiple tumors (syngeneic models)	Anti-tumor T-cell response	Gal-1 in tumor cells inhibits T cell activation and survival → immune privilege	Gal-1 blockade *in vivo* promotes tumor rejection and generates durable tumor-specific T cell memory.	(140)
	AML/ALL (myeloid leukemia)	CAR-T therapy	Leukemia-derived Gal-1 → CAR receptor downregulation on T cell surface → ↓cytotoxicity	Anginex (Gal-1 peptide inhibitor) rescues CAR-T killing. CD33 CARKR mutant resists Gal-1-mediated CAR loss.	(141)
	Ovarian Cancer (EOC)	Cisplatin/Paclitaxel (immune-mediated)	SNHG22 → miR-2467 sponge → ↑Gal-1 → PI3K/AKT + ERK activation	Gal-1 axis mediates immune-evasion component of chemo-resistance in EOC.	(130)
	Lung Cancer (NSCLC)	Anlotinib (immune context)	Gal-1 OE in fibroblasts → CAF phenotype → ↑tumor immune evasion and drug resistance	CAF-derived Gal-1 creates immunosuppressive niche; reduces T cell-mediated tumor clearance.	(131)
Galectin-3	Multiple cancers (biochemical/*in vivo*)	Anti-PD-1 (pembrolizumab) Anti-PD-L1 (atezolizumab)	Gal-3 potentiates PD-1/PD-L1 complex → physically blocks ICI antibody binding	GB1211 (Gal-3 inhibitor) restores ICI binding and ↑TILs; GB1211 + anti-PD-L1 reduces tumor growth *in vivo*.	(142)
	Melanoma/HNSCC	Belapectin + pembrolizumab (Phase I, NCT02575404)	Gal-3 blockade with belapectin (GR-MD-02) enhances anti-PD-1 activity	ORR: 50% MM, 33% HNSCC. Responders: ↑effector memory T cells, ↓M-MDSCs. No dose-limiting toxicity.	(143)
	Preclinical (cardio-oncology model)	Anti-CTLA-4/Anti-PD-1 (ICI cardiotoxicity)	Short-term ICI → ↑myocardial Gal-3 + NLRP3 + MyD88 → fibrosis, ↑IL-1β, ↑TNF-α, ↑SDF-1	Gal-3 upregulation is a putative biomarker of ICI-related myocardial and vascular damage.	(144)
	HCC	Atezolizumab + Bevacizumab (ATZ/BVZ)	Gal-4 → stabilizes LDHA → ↑glycolysis → HIF-1α → ↑CXCL6 → PD-L1+ neutrophil accumulation	Elevated serum Gal-4 predicts ATZ/BVZ resistance. Gal-4 KD restores CD8+ T cell function and ICI sensitivity.	(145)
Galectin-9	B-ALL	CAR-T therapy	MSC-secreted Gal-9 binds TIM-3 on CAR-T cells → ↓proliferation, ↓cytotoxicity	Gal-9 KD in MSCs restores CAR-T anti-tumor function; Gal-9/TIM-3 axis drives post-CAR-T relapse in B-ALL.	(146)

### 6.1 Galectins as modulators of therapeutic resistance and chemosensitivity

The mechanistic diversity of galectins documented in preceding sections, spanning immune evasion, myeloid reprogramming, metabolic adaptation, and stromal crosstalk, converges, in the clinical context, on a single critical challenge: therapeutic resistance. This section addresses how galectins actively modulate sensitivity to cytotoxic chemotherapy, targeted agents, and hormonal therapies across multiple cancer types, and examines the mechanistic pathways through which this resistance is established and potentially reversed. Gal-1 is the most extensively implicated galectin in chemoresistance. In HCC, Gal-1 overexpression protected cells from doxorubicin (DOX)- and sorafenib-induced death by activating PI3K signaling to upregulate P-glycoprotein (P-gp), an ATP-dependent drug efflux pump that reduced intracellular DOX accumulation; loss-of-function experiments confirmed P-gp as the obligate mediator of Gal-1-driven DOX resistance, but not sorafenib resistance, in P-gp-expressing cells, while cells lacking P-gp were unaffected, a mechanistic distinction with direct implications for patient stratification (126). In gastric cancer, CAF-secreted Gal-1 activated the NRP-1/c-JUN/Wee1 pathway to promote proliferation, metastasis, and cisplatin resistance; the NRP-1 inhibitor EG00229 effectively reversed these effects *in vitro*, and Gal-1 expression correlated with poor prognosis and NRP-1/Wee1 co-expression in clinical specimens (129). In esophageal squamous cell carcinoma (ESCC), serum Gal-1 levels were elevated in paclitaxel-resistant patients and correlated with treatment outcomes; Gal-1 stabilized Wnt/β-catenin signaling by promoting nuclear β-catenin accumulation, which increased MDR1 transcription and maintained cancer stem cell properties; specific clearance of Gal-1 from resistant patient serum restored paclitaxel sensitivity, and the pharmacological inhibitor OTX008 combined with taxanes effectively reversed resistance *in vitro* and in patient-derived xenografts (128). In enzalutamide-resistant prostate cancer, Gal-1 was significantly upregulated; its knockdown suppressed androgen receptor (AR) full-length and AR-V7 expression and their transcriptional activity, resensitizing cells to enzalutamide; the Gal-1 inhibitor LLS30 synergized with enzalutamide to inhibit resistant xenograft growth *in vivo*, identifying the Gal-1/AR/AR-V7 axis as a therapeutically tractable resistance mechanism (127).

In epithelial ovarian carcinoma (EOC), the lncRNA SNHG22 acted as a molecular sponge for miR-2467, releasing Gal-1 mRNA from suppression and activating PI3K/AKT and ERK cascades to confer resistance to both cisplatin and paclitaxel; SNHG22 knockdown increased chemosensitivity, establishing a non-coding RNA-mediated upstream regulatory layer for Gal-1-driven resistance (130). Gal-1 also contributes to resistance through stromal reprogramming: overexpression in normal lung fibroblasts converted them to CAFs whose conditioned medium decreased lung cancer cell apoptosis, increased Bcl-2/Bax ratio, and reduced sensitivity to anlotinib, implicating paracrine Gal-1 as a microenvironmental resistance factor independent of direct tumor cell expression (131).

Gal-3 mediates resistance through parallel pathways. In HER2-positive breast cancer, Gal-3 promoted resistance to trastuzumab by activating PI3K/AKT and Notch1 signaling to enhance cancer stemness; LGALS3 knockout had synergistic therapeutic effects with trastuzumab *in vivo* (132). In CRC, the BAP31/Gal-3/Wnt/β-catenin axis regulated 5-fluorouracil (5-FU) sensitivity: BAP31 knockdown reduced Gal-3 expression, inhibited β-catenin nuclear accumulation and downstream c-MYC/SOX2 transcription, decreased stemness, and resensitized cells to 5-FU; intrabodies against BAP31 enhanced 5-FU efficacy *in vivo* (133). In ovarian cancer, DMBT1 suppressed progression and cisplatin resistance by binding and downregulating Gal-3, inhibiting PI3K/AKT phosphorylation; Gal-3 overexpression reversed these effects, supporting Gal-3 as the downstream effector (135).

**Figure 5:**
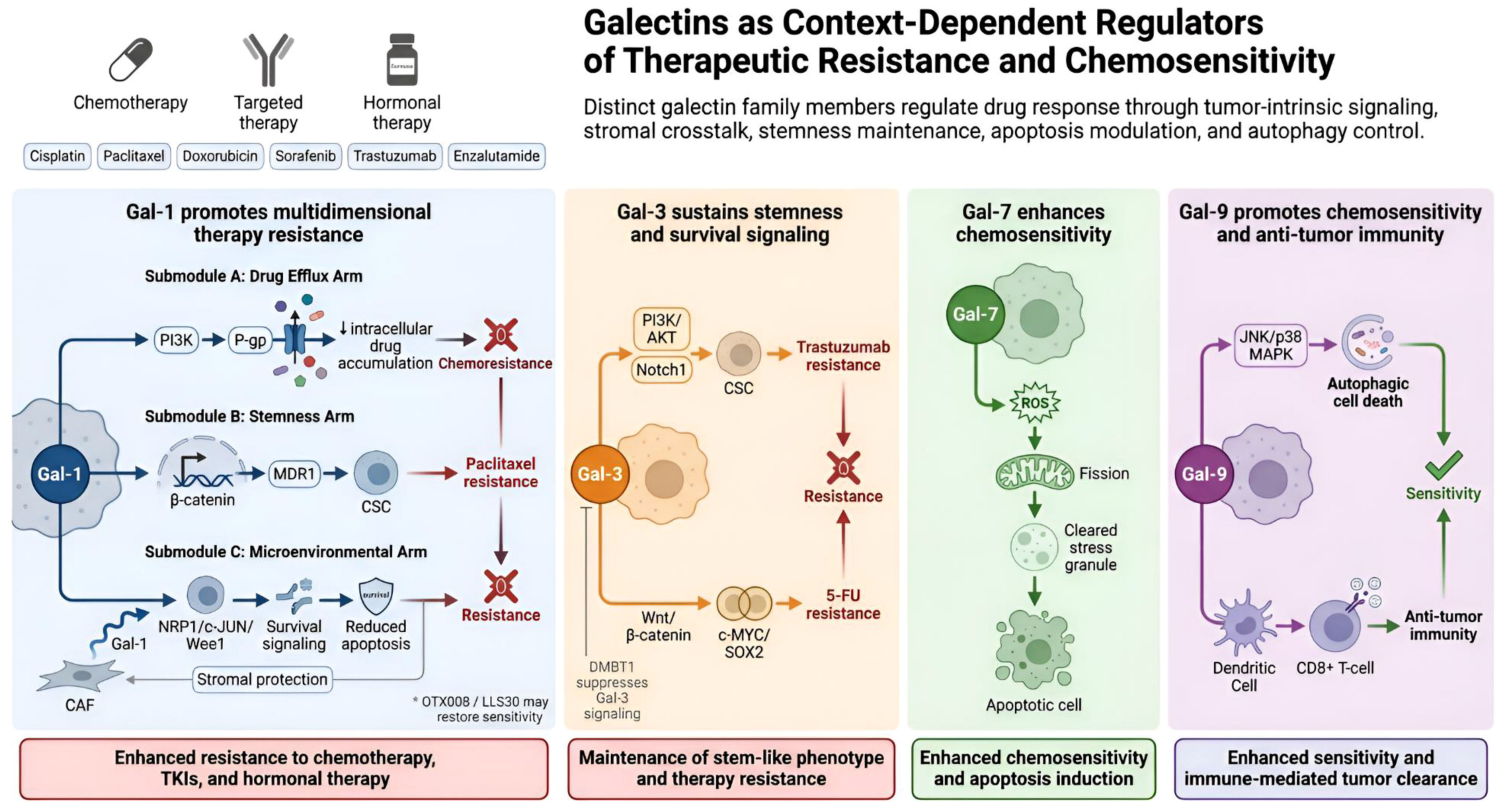
Galectins as context-dependent regulators of therapeutic resistance and chemosensitivity. Gal-1 drives resistance to doxorubicin, sorafenib, cisplatin, paclitaxel and enzalutamide via PI3K/P-gp drug efflux, Wnt/β-catenin-mediated stemness and CAF-dependent stromal protection. Gal-3 sustains stemness and survival signaling (PI3K/AKT/Notch1 and Wnt/β-catenin/c-MYC/SOX2) to confer trastuzumab and 5-FU resistance, whereas Gal-7 and Gal-9 enhance chemosensitivity through ROS/mitochondrial fission/apoptosis and autophagy inhibition plus anti-tumor immunity, respectively.

A contextual contrast is provided by two studies demonstrating pro-sensitivity or hepatoprotective roles of galectins. In cisplatin-treated rats, MCP-mediated Gal-3 inhibition exacerbated hepatotoxicity by increasing oxidative stress, malondialdehyde, and pro-inflammatory cytokines (IL-1β, TNF-α), indicating that hepatic Gal-3 serves a cytoprotective function against cisplatin-induced liver damage, a consideration that complicates systemic Gal-3 inhibitor strategies (134). In cervical cancer, Gal-7 promoted cisplatin-induced apoptosis by upregulating cleaved-PARP1, generating ROS, promoting mitochondrial fission, and facilitating stress granule clearance through the Gal-7/RACK1/G3BP1 axis, thereby reducing chemoresistance (136). Finally, Gal-9 exhibited non-apoptotic, caspase-independent cytotoxicity toward AML cells, including CD34^+^ patient-derived stem cells and cytarabine (AraC)-resistant lines, through autophagy inhibition, while sparing healthy cord blood-derived stem cells; Gal-9 also potentiated the effect of azacytidine, supporting its potential as a therapeutic agent in chemotherapy-refractory AML (137). Collectively, these studies reveal that galectins function as multi-pathway determinants of therapeutic response, and that their inhibition or activation, depending on tumor type and galectin identity, constitutes a rational strategy for overcoming treatment resistance (**Figure 5**).

### 6.2 Immunotherapy and CAR-T resistance

Gal-3 has been identified as a direct mediator of resistance to immune checkpoint inhibitors by potentiating the PD-1/PD-L1 interaction and physically blocking the binding of pembrolizumab and atezolizumab to their targets (142). Selective inhibition of Gal-3 with the small-molecule GB1211 restored checkpoint inhibitor binding in biochemical assays and reduced tumor growth in syngeneic murine models while increasing tumor-infiltrating T lymphocytes (142). Consistent with this, the foundational rationale for combining galectin-3 blockade with checkpoint inhibition was established earlier, when the orally active galectin-3 antagonist GB1107 was shown to reduce lung adenocarcinoma growth, increase intratumoral CD8^+^ T-cell infiltration, and augment the efficacy of anti–PD-L1 therapy in murine models (147). Clinical evidence from a phase I study of belapectin, a Gal-3 inhibitor, combined with pembrolizumab in patients with metastatic melanoma and head and neck squamous cell carcinoma showed objective response rates of 50% and 33%, respectively, with a favorable safety profile and fewer immune-mediated adverse events than expected with pembrolizumab monotherapy (143). Responding patients showed increased effector memory T cells, reduced monocytic myeloid-derived suppressor cells, and higher baseline Gal-3 tumor expression (143). In preclinical cardio-oncology models, short-term ICI therapy with anti-PD-1 or anti-CTLA-4 agents elevated myocardial Gal-3, fibrosis markers, and systemic inflammatory cytokines, suggesting Gal-3 also as a biomarker of ICI-related cardiotoxicity (144).

Gal-1 suppresses T-cell-mediated anti-tumor immunity and constitutes a key mechanism of tumor immune privilege; its *in vivo* blockade in tumor cells markedly enhanced T cell-mediated rejection and induced durable anti-tumor immune memory in syngeneic models (140). In NSCLC patients receiving anti-PD-1 therapy, elevated plasma and tissue Gal-1 levels were associated with a trend toward shorter progression-free survival, and *in vitro* BiTE assays confirmed stronger T-cell cytotoxicity against Gal-1-knockout tumor cells (138). In renal cell carcinoma treated with IO/TKI combinations, high Gal-1 expression independently predicted shorter progression-free survival (HR 2.505; p = 0.026), correlated with exhausted CD8+ T cells, elevated Tregs, and increased fibroblasts, and identified a patient subgroup deriving no benefit from IO/TKI therapy (139).

Within the CAR-T cell resistance landscape, Gal-1 secreted by myeloid leukemia cells was found to induce surface CAR downregulation under low effector-to-tumor ratio conditions, impairing cytotoxicity; the Gal-1-specific peptide anginex rescued killing activity *in vitro*, and a lysine-to-arginine mutant CAR construct (CARKR) demonstrated resistance to Gal-1-mediated downregulation (141). In B-ALL, bone marrow mesenchymal stromal cells suppressed CAR-T proliferation, upregulated inhibitory TIM-3, and reduced cytolytic function through soluble galectin-9 production induced by CAR-T-derived IFN-γ and TNF-α; shRNA knockdown of Gal-9 in stromal cells significantly restored CAR-T activity, identifying the Gal-9/TIM-3 axis as a therapeutically targetable resistance node (146).

**Figure 6:**
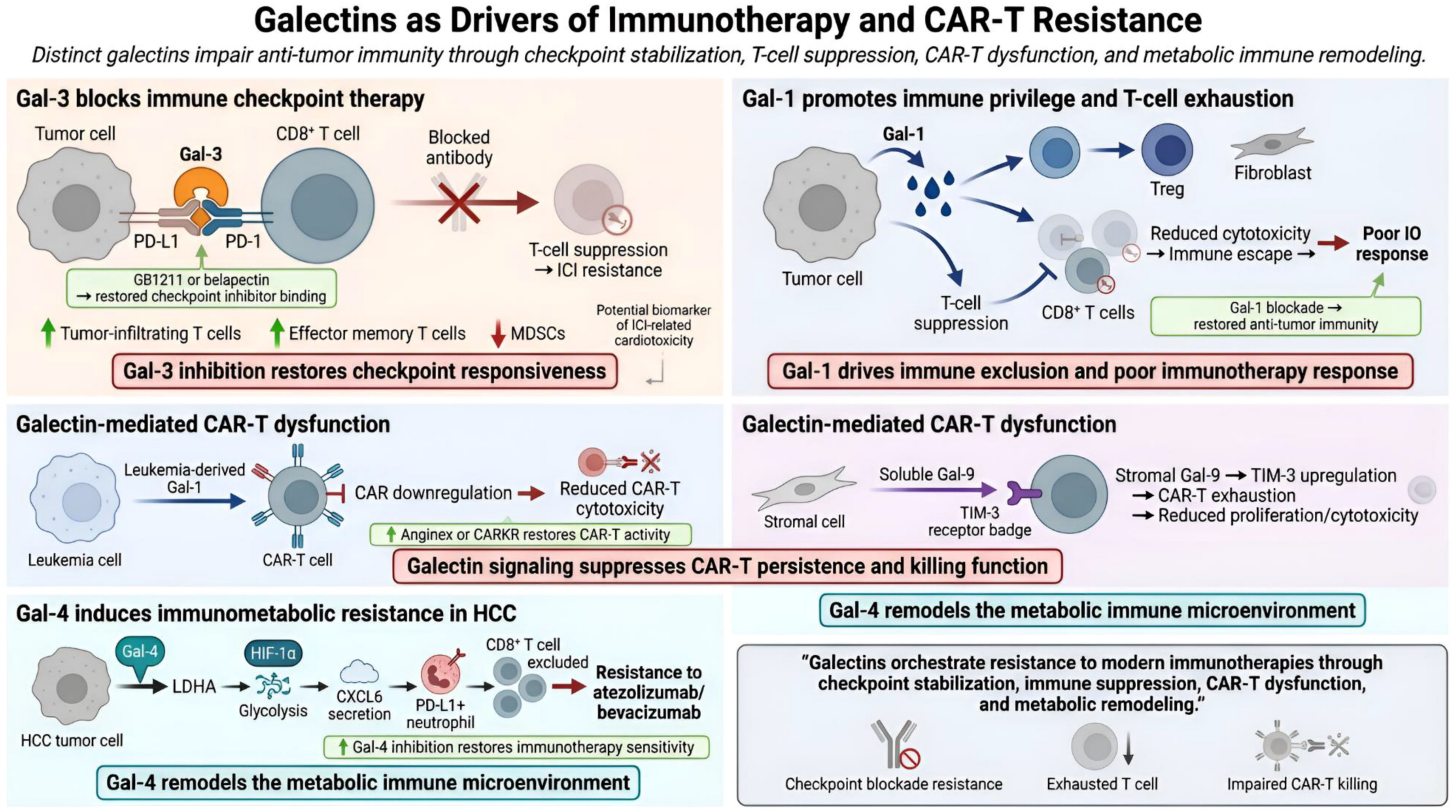
Galectins as drivers of immunotherapy and CAR-T resistance. Distinct galectins impair anti-tumor immunity through checkpoint stabilization (Gal-3), T-cell suppression and exhaustion (Gal-1), CAR-T cell dysfunction (Gal-1/Gal-9), and metabolic remodeling of the tumor microenvironment (Gal-4), leading to resistance to immune checkpoint inhibitors and CAR-T therapy.

In HCC, elevated serum Gal-4 correlated with primary resistance to atezolizumab/bevacizumab therapy and worse prognosis. Gal-4 stabilized LDHA by inhibiting TRIM28-dependent proteasomal degradation, amplifying glycolysis and HIF-1α-driven CXCL6 expression, which in turn promoted accumulation of immunosuppressive PD-L1+ tumor-associated neutrophils and reduced CD8+ T cell infiltration (145). Pharmacological or genetic inhibition of Gal-4 reversed these effects and restored sensitivity to combination immunotherapy in preclinical models, supporting the view that Gal-4 may function as both a resistance-associated factor and a candidate predictive biomarker in HCC (145) (**Figure 6**).

## 7 Emerging therapeutic strategies

The mechanistic evidence linking galectins to tumor progression and treatment resistance has motivated a growing translational effort to develop galectin-directed therapies. This section surveys current pharmacological strategies including small-molecule inhibitors, natural compounds, and pathway-targeted approaches, followed by nanomedicine and immunotherapeutic platforms integrating antibodies, nanoparticles, gene editing, and theranostic systems, and concludes with vaccination-based and combination immunotherapy strategies (**Table 4**).

**Table 4:** Galectin-targeted therapeutic strategies: small-molecule inhibitors, antibody-based approaches, nanomedicine platforms, and vaccine/epigenetic induction across cancer types.

Galectin	Cancer/application	Strategy/agent	Key mechanism	Key finding	Ref.
Small-molecule & natural compound inhibitors					
Galectin-3	Multiple cancers	K2 & L2 — synthetic non-carbohydrate small molecules	Bind Gal-3 CRD S-face → conformational change → inhibits adhesion, invasion, angiogenesis	K2/L2 reduce tumor growth and metastasis in vivo (mice & chick embryo); no cytotoxicity or genotoxicity detected.	(148)
	Cisplatin toxicity model (cardiac/renal)	Modified citrus pectin (MCP) — oral Gal-3 inhibitor	MCP ↓renal Gal-3 protein; dysregulates mitochondrial OXPHOS in cardiac and renal tissue under cisplatin	MCP + cisplatin ↑MDA, ↑IL-1β, ↑TNF-α in cardiac and renal tissue; Gal-3 plays a tissue-protective role in acute cisplatin toxicity.	(149)
	Thyroid Cancer	GB1107 & TD139 — small-molecule Gal-3 inhibitors	Inhibit AKT phosphorylation + ↓β-catenin + ↓MMP2 → counteract anoikis resistance	Both agents suppress migration and invasion dose-dependently; TD139 (high dose) induces apoptosis via caspase-3/PARP1.	(150)
	Cervical Cancer	Bergenin — natural polyphenol (C-glycoside)	Proteomic analysis: bergenin ↓Gal-3 and MMP-9 → anti-angiogenic effect	Bergenin reduces key angiogenic proteins; molecular docking confirms direct MMP-9 > Gal-3 binding affinity.	(151)
	Melanoma	Vemurafenib (PLX4032) + endogenous Gal-3 status	Gal-3 is a negative regulator of autophagy; Gal-3low cells show higher LC3-II accumulation and ER stress (↑GRP78)	Gal-3low/neg cells are more resistant to BRAFᵜᵜᵜᵜᵜ inhibition; pharmacological autophagy blockade (bafilomycin A) reverses this resistance.	(152)
	Breast/Ovarian Cancer (MUC16+); Multiple cancers	HPMA glycopolymers — polymer-based Gal-3 inhibitors	Competitive inhibition of Gal-3 CRD → blocks Gal-3-induced monocyte apoptosis and Gal-3–IFN-γ binding	Glycopolymers fully prevent Gal-3-induced M1 macrophage apoptosis and restore IFN-γ activity; promote M2→M1 macrophage repolarization.	(153)
Galectin-4	Gastric Cancer (Peritoneal metastasis)	Fucoidan analogs — chemically synthesized sulfated polysaccharides	Analog 10 (2,3-O-sulfated α(1,4)-L-fucose) binds Gal-4 Arg-45 → inhibits Gal-4 binding and cancer cell proliferation	Analog 14 (cholestanol-conjugated) shows enhanced inhibitory activity; fucoidan analogs suppress gastric cancer cell growth via Gal-4 pathway.	(154)
Galectin-1	Liver Cancer	JNK/c-Jun-ATF2 inhibitor — small molecule/genetic	CDDP activates JNK → phospho-c-Jun/ATF2 heterodimer → ↑Gal-1 → cisplatin resistance	JNK inhibition ↑DNA damage and overcomes cisplatin resistance in vitro and in vivo; dynamic bioluminescence imaging tracks JNK/Gal-1 activity in real time.	(155)
	Ovarian/Breast Cancer	Ganoderic acid T (GAT) — Ganoderma triterpenoid	GAT induces ubiquitination and degradation of Gal-1; ↓α-SMA+ cells, ↑TILs, ↑intratumoral drug concentration	GAT + paclitaxel ↑anti-tumor efficacy; GAT + anti-PD-L1 ↑CD8+ T cells in syngeneic mammary model; direct GAT–Gal-1 interaction by molecular docking.	(156)
Monoclonal antibodies, combination immunotherapy & vaccine strategies					
Galectin-1	Prostate Cancer	LLS30 (Gal-1 inhibitor) + anti-PD-1	LLS30 binds Gal-1 CRD → disrupts Gal-1–CD45 binding → ↓T-cell apoptosis → ↑intratumoral T-cell infiltration	LLS30 significantly improves anti-PD-1 efficacy in vivo; RNA-seq reveals novel tumor-intrinsic oncogenic mechanism of LLS30.	(157)
	Melanoma	Recombinant Gal-1 vaccine (bacterial fusion protein)	Anti-Gal-1 antibodies generated by vaccination impair tumor angiogenesis and reduce immunosuppression	Vaccination against Gal-1 impairs melanoma growth; ↑tumor perfusion, ↑CTL and macrophage infiltration, ↑granzyme B; correlates with reduced tumor burden.	(158)
	Prostate Cancer	Gal-1 inhibition (siRNA or LLS30) + cancer vaccine (combined strategy)	Low-dose docetaxel ↓Gal-3 expression in PCa cells → enables vaccine-induced Th1 response and CD8+ T-cell proliferation	Gal-3 in PCa cells is the primary immune checkpoint blocking vaccine efficacy; low-dose docetaxel pre-conditioning + vaccination significantly controls tumor recurrence.	(159)
Galectin-3	PDAC	Anti-Gal-3BP monoclonal antibody (2 clones)	Gal-3BP enhances Gal-3–EGFR signaling → ↑cMyc + EMT; antibody blocks Gal-3BP-mediated metastatic cascade	Gal-3BP identified by LC-MS/MS as highly secreted in PDAC; antibody clones profoundly abrogate PDAC metastasis in vivo.	(160)
	Sarcoma/Mammary/Prostate cancer (murine)	Belapectin (Gal-3 inhibitor) + agonist anti-OX40 antibody	Gal-3 blockade + OX40 co-stimulation → ↑CD8+ TILs, ↓Tregs, ↓M-MDSCs, restored iNOS/arginase-1 balance	Combination synergizes for tumor regression and survival in 3 models via CD8+ T-cell-dependent mechanism.	(161)
	Ovarian Cancer (high-grade serous, MUC16+)	Anti-Gal-3 monoclonal antibody 14D11 (CRD-directed)	14D11 competes with lactose for Gal-3 CRD → blocks AKT/ERK1/2 phosphorylation → ↓Matrigel invasion	14D11 prolongs OS in flank tumor models and retards lung metastatic growth in MUC16-expressing breast cancer.	(162)
	Melanoma/Breast Cancer (TCGA; Gal-3-specific T cells)	Gal-3-derived long peptide therapeutic vaccine	Peptide epitope expands Gal-3-specific CD8+ T cells that recognize and eliminate Gal-3-expressing TME cells	Vaccination delays tumor growth, ↓Tregs and Gal-3+ non-myeloid CD45+ cells in tumor; spontaneous Gal-3-specific T-cell responses found in healthy donors and cancer patients.	(163)
Galectin-9	PDAC (early neoplastic lesions)	Anti-Gal-9 monoclonal antibody (patented, EP3152234B1)	Anti-Gal-9 mAb neutralizes Gal-9 on Tregs → ↓Treg suppressive activity → ↓PanIN progression	First demonstration of anti-Gal-9 mAb limiting PanIN progression in KC transgenic mice; Gal-9 and Tregs increase proportionally with PanIN grade.	(164)
	Lung/Colorectal Cancer	Anti-Gal-9 antibody + EGFR-TKI (afatinib/osimertinib)	EGFR-TKI → DNA damage → cGAS-STING-IFN-I → ↑Gal-9 in tumor + myeloid cells → immune escape	Anti-Gal-9 + EGFR-TKI remodels myeloid landscape toward antigen-presenting DCs and ↑CD8+ T-cell responses; active even in poorly immunogenic LLC model unresponsive to PD-1/PD-L1.	(165)
	Multiple cancers (syngeneic murine models)	Anti-Gal-9 antibody + ATM inhibitor (AZD1390)	ATM inhibition → cGAS-STING-IFN-β → ↑Gal-9 secretion; Gal-9 neutralization reverses immune escape	Combination significantly suppresses tumor growth and prolongs survival; active in LLC tumors unresponsive to PD-1/PD-L1; ATM KO potentiates anti-Gal-9 efficacy.	(166)
	Breast Cancer (anthracycline-treated)	Anti-Gal-9 therapy + doxorubicin	Doxorubicin → STING/IFN-β → ↑tumoral Gal-9 → immune escape; anti-Gal-9 blocks this counter-response	Combining doxorubicin + anti-Gal-9 significantly inhibits tumor growth and prolongs OS in syngeneic models; p-STING/Gal-9 elevated in anthracycline-treated breast cancer patient tissues.	(167)
	PDAC	Anti-Gal-9 antibody + anti-PD-L1 antibody	Dual blockade of Gal-9/TIM-3 and PD-1/PD-L1 axes concurrently	Gal-9 and PD-L1 co-overexpressed in human PDAC; combined anti-Gal-9 + anti-PD-L1 superior to either monotherapy in murine PDAC model.	(22)
Nanomedicine, siRNA delivery & gene editing platforms					
Galectin-1	Solid tumors (general)	APN — antibody-like polymeric nanoparticle with galactose recognition units + Tuftsin	Galactose units capture intratumoral Gal-1; Tuftsin activates macrophage-mediated phagocytosis of Gal-1 complexes	APN depletes intratumoral Gal-1 and restores TIL anti-tumor activity; modular platform adaptable to other immunosuppressive target proteins.	(168)
	Cervical Cancer (lymph node metastases)	TDG@PMI NPs — Gal-1-targeted PEG@MnPb-IR820 nanoparticles for MRI/NIR/PTT	Gal-1-targeting → selective LN accumulation (74 nm); NIR laser irradiation → mitochondrial apoptosis in metastatic cells	Nanoprobe enables dual-modal MRI+NIR imaging of LN metastases; single NIR laser irradiation achieves anti-tumor PTT effect in sentinel LNs in vivo.	(169)
	Cancer diagnostics (theranostic nanovector)	GEM@DOX gold nanovector (PEG-AuNPs) with Gal-1 targeting for drug delivery and biomarker detection	Gal-1 conjugated to GEM@DOX in PEG-AuNPs detects cancer cells via SPR and Raman shift correlated with Gal-1 concentration	Theranostic hybrid nanovector with dual chemo-delivery (DOX + GEM) and Gal-1-based cancer cell detection; Raman/SPR spectroscopy confirms Gal-1 interaction.	(170)
Galectin-3	Multiple cancers (theranostics)	Anti-Gal-3 mAbs (QMAb-1/2) labeled with ^89^Zr (PET) and ^177^Lu (radioimmunotherapy)	QMAb-1 shows superior tumor uptake and retention; minimal off-target accumulation; ^177^Lu-DTPA-QMAb-1 delivers targeted radiotherapy	[^89^Zr]QMAb-1 enables PET/CT imaging stratified by Gal-3 expression; [^177^Lu]QMAb-1 shows potent anti-tumor efficacy without hepatotoxicity; validated in TNBC models.	(171)
Galectin-9	Melanoma	FSGG/siGal-9 — transdermal photothermal nanosensitizer loading Gal-9 siRNA	Gal-9 knockdown blocks Gal-9/TIM-3 axis; synergizes with PTT to prevent CTL exhaustion	Gal-9 identified as the dominant immune checkpoint on solid tumors (> PD-L1/CD80); FSGG/siGal-9 + PTT inhibits tumor growth and enhances anti-tumor CTL immunity.	(172)
	Glioblastoma (GBM)	T7-Exo/siGal-9 — T7 peptide-decorated exosomes electroporated with Gal-9 siRNA	siGal-9 delivery → ↓Gal-9 in GBM → activates TLR7-IRF5 pathway → M1 macrophage polarization → ↑CD8+ T-cell cytotoxicity	T7-Exo/siGal-9 promotes macrophage M2→M1 repolarization and enhances GBM phagocytosis; demonstrated in xenograft C57BL/6 models.	(173)
	Multiple cancers (primary + distant tumors)	CaP NP — calcium phosphate nanoparticle CRISPR-Cas9 dual knockout of PD-L1 + Gal-9	Intratumoral RNP delivery → knockout PD-L1 and Gal-9 simultaneously; Ca^2+^ overload triggers DAMP release → amplifies anti-tumor immunity	Dual KO evokes local and systemic anti-tumor responses; reduces lung metastasis by converting circulating tumor cells to PD-L1^-^/Gal-9^-^ phenotype.	(174)
	PDAC	iEXO-OXA — BM-MSC exosomes with Gal-9 siRNA + surface oxaliplatin prodrug	Gal-9 siRNA disrupts Gal-9/Dectin-1 axis → M2→M1 TAM repolarization; OXA induces ICD → ↑CTL recruitment + ↓Tregs	Dual-action system (immunogenic cell death + Gal-9 silencing) achieves significant therapeutic efficacy in PDAC; BM-MSC exosomes improve tumor targeting.	(175)
Galectin induction & nanobody-based imaging					
Galectin-7	Colorectal Cancer (MSS, immune-cold)	EHMT2 inhibition → epigenetically induced Gal-7 upregulation	EHMT2/CHD4 silencing complex represses Gal-7; EHMT2 inhibition de-represses Gal-7 → T-cell-mediated cytotoxicity activation	EHMT2 inhibition converts cold MSS TME to T-cell-inflamed; Gal-7 administration enhances anti-PD-1 efficacy in MSS CRC as adjunct cytokine.	(176)
	Triple-Negative Breast Cancer (TNBC)	G7N8 nanobody (Gal-7-specific) conjugated with ^64^Cu-NOTA for PET imaging	G7N8 binds Gal-7 with high affinity (no cross-reactivity to Gal-1/-3/-8/-9); inhibits Gal-7 binding to T-cell glycoreceptors → ↓T-cell apoptosis	^64^Cu-NOTA-G7N8 achieves selective radiotracer accumulation in TNBC tumors at 20 h PET imaging; first Gal-7-specific tool for immunotherapy and molecular imaging.	(177)

### 7.1 Galectin-targeted therapeutic strategies in cancer

Galectins are increasingly recognized as important modulators of tumor progression, immune evasion, and drug resistance, which has made them subjects of growing therapeutic interest in oncology. A growing body of research has focused on developing inhibitory strategies against various galectin family members, with encouraging preclinical findings and early-phase clinical signals. A substantial part of this effort builds on rationally designed glycomimetic inhibitors developed by Nilsson, Leffler and colleagues. Early thiodigalactoside (TDG) diester derivatives provided proof of concept, inhibiting galectin-1, -3, -7 and -9 in the low-micromolar range and exerting antimigratory effects on lung and prostate cancer cells (178). Iterative optimization of these scaffolds subsequently yielded orally available, sub-micromolar glycomimetics with defined selectivity, including the galectin-1-selective inhibitor GB1908 (179) and the dual galectin-1/galectin-3 inhibitor GB1841 (180), both of which suppressed Lewis lung carcinoma growth in syngeneic models, as well as the galectin-3-selective tool compound GB2095, which reduced breast and melanoma tumor growth *in vivo* (181). Beyond therapeutic candidates, domain-selective ligands have provided valuable chemical probes; for example, epimeric galactoside and guloside derivatives discriminate between the C- and N-terminal carbohydrate-recognition domains of galectin-9, enabling dissection of domain-specific functions (182). Importantly, this medicinal-chemistry program has advanced into the clinic: the orally bioavailable galectin-3 inhibitor GB1211 showed favorable safety and pharmacokinetics in a first-in-human phase 1 study (NCT03809052) (183) and has since progressed to phase 2 evaluation (181). Among the most extensively studied targets, Gal-3 has been the focus of multiple inhibitor development programs. Sindrewicz-Goral et al. (148) reported the discovery of two novel non-carbohydrate small molecule inhibitors, K2 and L2, which bind to the canonical S-face of the Gal-3 carbohydrate-recognition domain (CRD). These compounds significantly inhibited cancer cell adhesion, invasion, angiogenesis, and pro-inflammatory cytokine secretion *in vitro*, while markedly reducing tumor growth and metastasis *in vivo* without detectable cytotoxicity or genotoxicity. In a complementary approach, Filipová et al. (153) developed HPMA-based glycopolymers bearing tetrasaccharide ligands that completely inhibited Gal-3-induced apoptosis of monocytes and macrophages, thereby preserving interferon-gamma function and supporting anti-tumor immunity. Furthermore, Lee et al. (150) demonstrated that the Gal-3 inhibitors GB1107 and TD139 suppressed anoikis resistance, motility, and invasive capacity in thyroid cancer cells through the AKT/β-catenin pathway. Santos et al. (149) investigated modified citrus pectin (MCP) as a Gal-3 inhibitor in cisplatin-induced cardiac and renal toxicity, revealing that while MCP reduced renal Gal-3 levels, it paradoxically increased oxidative stress, underscoring the need for careful evaluation of Gal-3 inhibition in different disease contexts.

Beyond galectin-3, other family members have also attracted therapeutic interest. Ji et al. (154)synthesized fucoidan analogs targeting Gal-4, demonstrating significant suppressive activity against gastric cancer cell proliferation. Molecular docking and mutagenesis studies identified Arginine-45 as critical for binding, highlighting specific structural determinants for inhibitor design. Sun et al. (176) uncovered a novel role for Gal-7, showing that EHMT2 inhibition upregulated Gal-7 expression, transforming the immunosuppressive microenvironment of microsatellite-stable colorectal cancer into a T-cell-inflamed state and enhancing anti-PD1 efficacy.

Gal-1 has also been implicated in drug resistance and immune modulation. Yang et al. (155) demonstrated that JNK/c-Jun-ATF2 signaling upregulates Gal-1 expression, mediating cisplatin resistance in liver cancer, and that JNK inhibition overcame this resistance both *in vitro* and *in vivo*. Chen et al. (156) showed that ganoderic acid T, a Ganoderma triterpenoid, downregulated Gal-1 through ubiquitination, modulating the tumor microenvironment and enhancing the efficacy of both chemotherapy and immunotherapy in ovarian and mammary cancer models.

Natural compounds have also shown promise in targeting galectins. Chauhan et al. (151) reported that bergenin reduced Gal-3 and MMP-9 expression, inhibiting angiogenesis in cervical carcinoma cells. Bustos et al. (152) revealed that Gal-3 negatively regulates autophagy in melanoma cells and that its loss promotes resistance to vemurafenib through enhanced autophagic flux, suggesting that autophagy inhibition could be a viable combination strategy. Collectively, these studies suggest that targeting galectins through diverse pharmacological approaches may have therapeutic value for modulating the tumor microenvironment, addressing selected forms of drug resistance, and enhancing the efficacy of existing cancer therapies.

### 7.2 Galectin-targeted nanomedicine and immunotherapeutic approaches in cancer

The convergence of nanotechnology with galectin-targeted strategies has expanded the range of approaches being explored for cancer therapy and diagnostics. Recent advances encompass antibody-based interventions, nanoparticle-mediated drug delivery, gene editing platforms, and theranostic systems, all aimed at disrupting galectin-mediated immune suppression and tumor progression.

Antibody-based strategies have demonstrated considerable promise across multiple galectin targets. Stasenko et al. (162) developed a high-affinity monoclonal antibody (14D11) targeting the galectin-3 carbohydrate-binding domain, which inhibited cancer cell invasion and prolonged survival in MUC16-expressing ovarian and breast cancer models. Choi et al. (160) identified galectin-3 binding protein (Gal-3BP) in PDAC and developed antibody clones that abrogated metastasis by disrupting Gal-3BP-mediated EGFR signaling. Sturgill et al. (161) showed that combining belapectin with anti-OX40 therapy synergistically promoted tumor regression through enhanced CD8+ T cell infiltration and reduced MDSC-mediated immunosuppression. For galectin-9, Quilbe et al. (164) developed a monoclonal antibody that limited pancreatic neoplastic lesion progression by targeting regulatory T cell suppressive activity. Linghu et al. (165) demonstrated that anti-galectin-9 therapy synergized with EGFR inhibitors to reprogram the tumor microenvironment, overcoming immune evasion in lung cancer models. Zheng et al. (166) revealed that galectin-9 blockade combined with ATM inhibition induced potent anti-tumor immunity via the cGAS-STING-IFNβ pathway.

Beyond conventional antibodies, novel molecular platforms have expanded the therapeutic repertoire. Nehmé et al. (177) developed Gal-7-specific nanobodies that inhibited T-cell apoptosis and enabled PET imaging in triple-negative breast cancer models, offering dual diagnostic and therapeutic potential. Gu et al. (168) engineered an antibody-like polymeric nanoparticle (APN) capable of specifically capturing and removing intratumoral Gal-1, thereby enhancing antitumor T-cell responses through macrophage-mediated phagocytosis.

Nanoparticle-mediated delivery systems have further advanced galectin-targeted therapy. Ren et al. (172) developed a transdermal photothermal nanodrug (FSGG/siGal-9) that knocked down Gal-9 expression in melanoma, blocking the Gal-9/TIM-3 axis and synergizing photothermal therapy with anti-tumor immunity. Li et al. (173) designed T7 peptide-decorated exosomes loaded with galectin-9 siRNA, promoting macrophage repolarization to the M1 phenotype and enhancing CD8+ T cell cytotoxicity against glioblastoma. Fang et al. (174) employed calcium phosphate nanoparticles for CRISPR-Cas9-mediated dual knockout of PD-L1 and Gal-9 in tumor cells, evoking robust local and systemic anti-tumor immune responses and reducing lung metastasis in mouse models.

Theranostic applications have also gained momentum. Duan et al. (169) developed a galectin-1-targeted nanoprobe enabling dual-modal MRI/NIR imaging and photothermal therapy for cervical cancer lymph node metastases, demonstrating excellent anticancer activity with low biological toxicity. Khan et al. (170) engineered gold-based nanovectors conjugated with galectin-1 for spectroscopic cancer diagnostics, combining doxorubicin and gemcitabine delivery with biomarker detection. Zhang et al. (171) developed galectin-3-targeted radioimmunoligands labeled with ^89^Zr and ^177^Lu, demonstrating potent radiotheranostic potential in triple-negative breast cancer models with minimal off-target toxicity. Collectively, these studies illustrate the breadth of galectin-targeted nanomedicine and immunotherapy strategies currently under development. The integration of antibody engineering, nanotechnology, gene editing, and theranostic platforms provides a diverse translational toolkit, although its clinical value remains to be established across specific tumor settings.

### 7.3 Galectin-targeted vaccination and combination immunotherapy strategies in cancer

Therapeutic vaccination and combination immunotherapy strategies targeting galectins are being explored as approaches to counter tumor-induced immune suppression. These strategies aim to activate anti-tumor immunity either by directly targeting galectin-expressing cells or by combining galectin inhibition with established immunotherapeutic modalities. Vaccination-based approaches have demonstrated efficacy against multiple galectin family members. Femel et al. (158) showed that vaccination against Gal-1 significantly impaired melanoma growth through improved vascular perfusion and increased cytotoxic T lymphocyte (CTL) infiltration. Bendtsen et al. (163) identified spontaneous T-cell responses against galectin-3-derived peptides and showed that therapeutic vaccination significantly delayed tumor growth in mammary cancer models by reducing regulatory T cells and Gal-3-positive cells in TME. Tiraboschi et al. (159) suggested that Gal-3 represents an important barrier to vaccine-based immunotherapy efficacy in their prostate cancer model, and that combining low-dose docetaxel with vaccination helped control tumor recurrence through enhanced CD8+ T cell infiltration.

Combination strategies integrating galectin blockade with checkpoint inhibitors and chemotherapy have yielded synergistic outcomes. Li et al. (22) demonstrated that combined anti-Gal-9 and anti-PD-L1 treatment achieved greater tumor growth reduction in pancreatic cancer than either therapy alone. Sun et al. (167) revealed that doxorubicin upregulated Gal-9 via the STING/IFNβ pathway, and combining anthracyclines with anti-Gal-9 therapy significantly prolonged survival. Wang et al. (157) showed that the galectin-1 inhibitor LLS30 suppressed T cell apoptosis and significantly improved anti-PD-1 efficacy in prostate cancer models.

**Figure 7:**
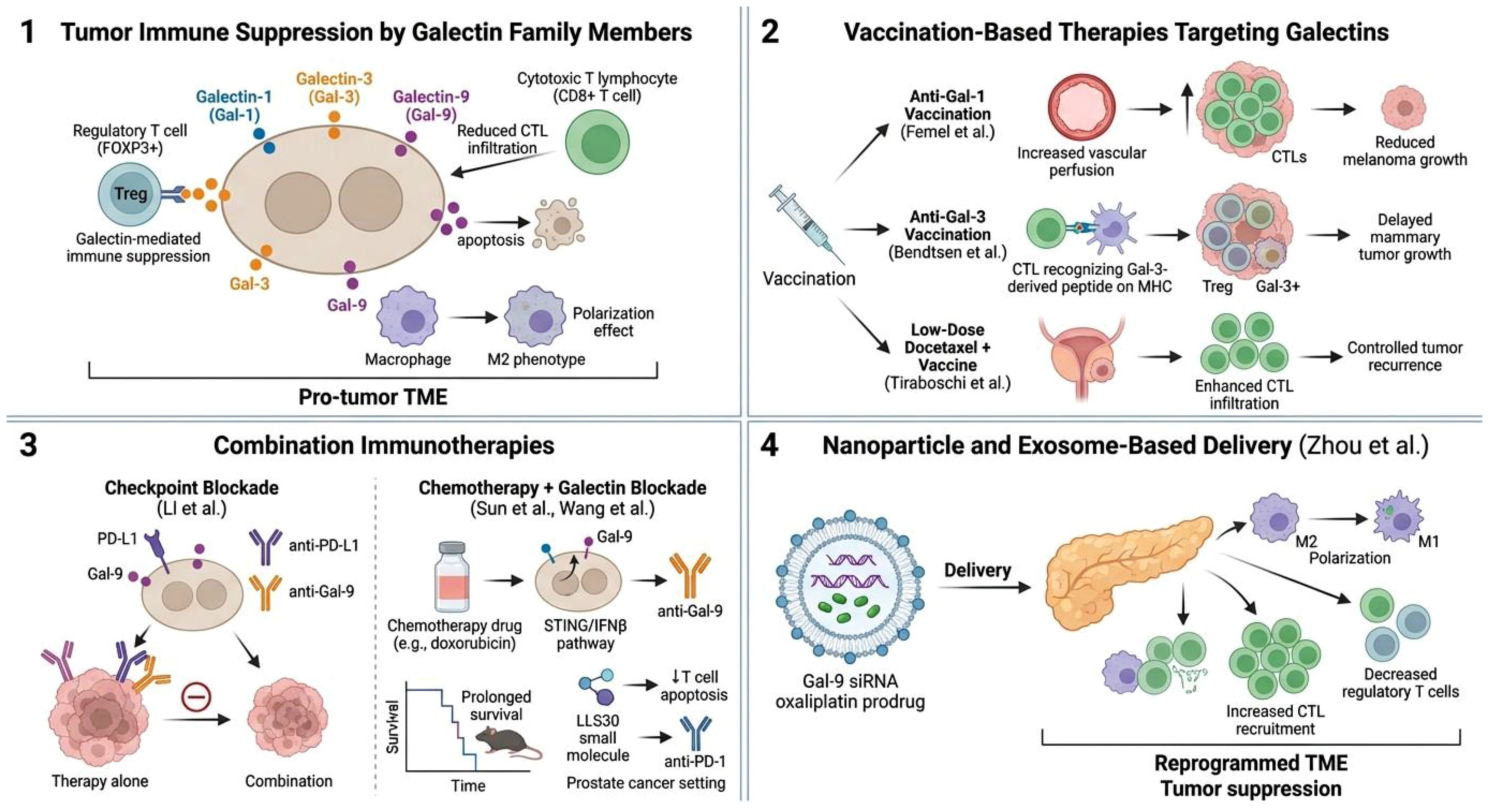
Schematic overview of emerging galectin-targeted therapeutic strategies in cancer. Current approaches include small-molecule and natural-compound inhibitors, antibodies, nanobodies, nanoparticles, gene-editing platforms, theranostic systems, and vaccination strategies designed to disrupt galectin-mediated tumor progression, immune suppression, and treatment resistance. These multimodal interventions, particularly when combined with checkpoint blockade, chemotherapy, or other immunotherapies, highlight galectins as promising translational targets for enhancing anti-tumor efficacy across diverse cancer settings.

Nanoparticle-based delivery has further enhanced these strategies. Zhou et al. (175) developed exosome-based dual delivery systems loaded with galectin-9 siRNA and oxaliplatin prodrug, which reprogrammed the pancreatic cancer TME by promoting macrophage polarization, recruiting cytotoxic T lymphocytes, and downregulating regulatory T cells. Collectively, these findings suggest that galectin-targeted vaccination and combination immunotherapy may represent useful strategies for enhancing anti-tumor immunity across multiple cancer types (**Figure 7**).

## 8 Galectins as diagnostic, prognostic, and predictive biomarkers in cancer

Galectins have gained increasing attention as candidate biomarkers in oncology, with circulating and tissue expression levels showing potential diagnostic, prognostic, and predictive value across a broad spectrum of malignancies (**Table 5**). Gal-1 has been consistently associated with tumor aggressiveness and poor clinical outcomes. Díaz Del Arco et al. (191) identified stromal Gal-1 overexpression as an independent prognostic factor for cancer-specific survival in gastric adenocarcinoma, correlating with advanced staging and recurrence. Similarly, Salunkhe et al. (194) demonstrated that Gal-1 expression increased with advancing histological grade and clinical stage in oral squamous cell carcinoma. In cervical cancer, Punt et al. (193) showed that strong tumor cell Gal-1 expression independently predicted poor survival. Masoodi et al. (187) reported that serum Gal-1 outperformed CA125 in distinguishing benign from malignant ovarian tumors, while Wu et al. (186) demonstrated that circulating Gal-1 predicted lymph node metastasis in colorectal cancer patients, even in those with normal carcinoembryonic antigen levels. Chen et al. (195) further revealed that Gal-1 promotes lung cancer cell survival by enhancing DNA repair through the PARP1/H1.2 interaction, and that its inhibitor thiodigalactoside restored sensitivity to etoposide. Liu et al. (185) developed a Gal-1-targeted PET radiotracer enabling noninvasive assessment of the immunosuppressive tumor microenvironment to predict immunotherapy responses.

**Table 5:** Galectins as diagnostic, prognostic, and predictive biomarkers in cancer.

Galectin	Cancer type	Sample/method	Key biomarker findings	Ref.
Gal-3	Lung adenocarcinoma	Serum (ELISA), tumorsphere, co-culture	Soluble Gal-3: independent prognostic biomarker for OS and RFS; AUC 0.801 for predicting pembrolizumab response	(184)
Gal-1	Multiple solid tumors	PET imaging (^124^I-αGal-1 radiotracer)	Gal-1-targeted PET predicted ICB resistance; Gal-1 inhibition remodeled immunosuppressive TME	(185)
Gal-1, Gal-3	Colorectal cancer	Serum (ELISA)	Gal-1 predicted lymph node metastasis even with normal CEA; 90K/Mac-2BP correlated with tumor size	(186)
Gal-1	Ovarian cancer	Serum (ELISA)	Gal-1 outperformed CA125 in distinguishing benign vs malignant; decreased post-surgery and chemotherapy	(187)
Gal-2	Breast cancer	mRNA (KM plotter), IHC	LGALS2 overexpression predicted better OS in all subtypes, especially luminal B and basal-like	(188)
Gal-3	Pancreatic cancer	Serum (time-resolved fluorescence immunoassay)	Biomarker for screening, early diagnosis, prognosis; high specificity for predicting 3-year survival	(189)
Gal-1, -3, -9	Breast cancer	Serum (ELISA), genetic mutation analysis	KIT mutations correlated with elevated Gal-9; TP53+PIK3CA mutations with elevated Gal-3	(190)
Gal-1	Gastric adenocarcinoma	Tissue (IHC, TMA)	Stromal Gal-1: independent prognostic factor for CSS; stratified stage III into prognostic subgroups	(191)
Gal-9	Triple-negative breast cancer	Tissue (IHC, TMA)	Low Gal-9 correlated with higher stage and LVI; high Gal-9 associated with sTILs and PD-L1 positivity	(192)
Gal-1, -3, -9	Squamous cervical cancer	Tissue (IHC, n=160)	Gal-1: independent predictor for poor survival (HR 8.02); Gal-3 correlated with invasion; Gal-9 trended toward improved survival	(193)
Gal-1	Oral squamous cell carcinoma	Tissue (IHC)	Expression increased with advancing histological grade and clinical stage	(194)
Gal-1	Lung cancer	Tissue, proteomics, mouse model	Gal-1 enhanced PARP1/H1.2-mediated DNA repair; TDG inhibitor restored etoposide sensitivity	(195)
Gal-3	Triple-negative breast cancer	Co-culture, RNA-seq, mouse model	Gal-3 induced T cell exhaustion (CD8/PD1/TIM3/LAG3+) and Treg upregulation via oxidative phosphorylation dysfunction	(196)
Gal-3	Triple-negative breast cancer	Tissue (IHC, n=489), mouse model	Co-expression of Gal-3 and vimentin promoted EMT, metastasis; lower Gal-3 associated with poor survival	(197)
Gal-7	Thymic epithelial tumors	Tissue (proteomics, IHC, n=164)	Gal-7 expressed in 82% high-risk vs 13% low-risk TETs; correlated with advanced stage and reduced PFS	(19)
Gal-3	Cervical cancer	Tissue (IHC), flow cytometry, mouse model	Gal-3 correlated with LN metastasis, recurrence; promoted MDSC recruitment via STAT3/Akt pathway	(67)
Gal-9	Small cell lung cancer	Tissue (IHC, n=102)	Gal-9-based immune risk score model (AUC 0.671) predicted relapse; high Gal-9 on TILs indicated better RFS	(198)
Gal-9	Intrahepatic cholangiocarcinoma	Serum (ELISA), tissue (IHC, n=91)	Circulating Gal-9 ≥12.0 ng/ml linked to poorer OS and RFS; correlated with glycolysis markers	(199)
Gal-1, -3, -9	Breast cancer	Serum (ELISA)	Gal-9 elevated in HER2-enriched; Gal-1 increased post-therapy; subtype-specific expression profiles	(200)
Gal-7, Gal-8	Breast cancer	Tissue (IHC, n=235)	High Gal-7: impaired PFS and DDFS; high Gal-8: improved OS; combination was strongest negative prognosticator	(201)
Gal-9	Hepatocellular carcinoma	Serum (ELISA), tissue (IHC), bioinformatics	Serum Gal-9: independent predictor of recurrence; reflected immune-evasive TME with CD163+ and FOXP3+ infiltrates	(202)
Gal-3	Colorectal carcinoma	Fecal and serum (ELISA)	Fecal Gal-3 correlated with nuclear grade, TNM stage, metastasis; positively correlated with AFP and CEA	(203)
Gal-3	Breast cancer	Plasma (ELISA), tissue (IHC, n=88)	Chemotherapy-induced Gal-3 increase: marker of efficacy; longer disease-free interval over 84-month follow-up	(204)

Gal-3 has shown potential biomarker utility in multiple cancer types. Yi et al. (189) showed that serum Gal-3 serves as a biomarker for screening, early diagnosis, and prognosis in pancreatic cancer. Torres-Martínez et al. (184) identified soluble Gal-3 as an independent prognostic biomarker in lung adenocarcinoma with high predictive efficiency for pembrolizumab response. In breast cancer, Shafiq et al. (204) demonstrated that chemotherapy-induced increases in extracellular Gal-3 correlated with longer disease-free intervals, while Raiter et al. (196) revealed that Gal-3 secreted by TNBC cells induces immunosuppression through exhausted CD8+ T cells and regulatory T cells. Ram et al. (197) showed that co-expression of Gal-3 and vimentin in TNBC promotes tumor progression. Jovanovic et al. (203) reported that fecal Gal-3 correlated with disease severity in colorectal carcinoma. Mai et al. (67) demonstrated that Gal-3 suppresses CD8+ T cell function via MDSC recruitment in cervical cancer.

Other galectin family members have also shown prognostic relevance. Galectin-9 expression has been associated with immune cell infiltration and clinical outcomes in TNBC (192), and elevated serum Gal-9 predicted early recurrence in HCC (202) and poor prognosis in intrahepatic cholangiocarcinoma (199). Chen et al. (198) developed a Gal-9-based immune risk score model for predicting relapse in small cell lung cancer. Chetry et al. (188) reported that galectin-2 overexpression predicts better prognosis in breast cancer, while Lv et al. (19) identified galectin-7 as a biomarker for aggressiveness in thymic epithelial tumors. Trebo et al. (201) showed that high Gal-7 combined with low galectin-8 expression independently predicted poor outcomes in breast cancer.

Multi-galectin profiling approaches have further enhanced biomarker utility. Funkhouser et al. (200) analyzed serum galectin-1, -3, and -9 patterns across breast cancer subtypes, revealing subtype-specific expression profiles. Markalunas et al. (190) demonstrated correlations between genetic mutations and galectin levels, identifying KIT mutations as correlating with elevated Gal-9 and TP53 mutations with higher Gal-3 in breast cancer. Collectively, these studies support the view that galectins may represent a multifaceted biomarker family with potential relevance to cancer diagnosis, prognosis, treatment selection, and therapeutic monitoring across diverse malignancies.

## 9 Limitations and future perspectives

While this review integrates mechanistic, preclinical, and translational findings across the galectin family, several limitations should be acknowledged. First, the majority of the evidence derives from preclinical models; for example, anti-Gal-9 therapy (26), Gal-13 mimetic peptides (99), and the Gal-3 inhibitor GB1211 (142), have been evaluated exclusively in syngeneic or xenograft systems. Clinical evaluation of selective galectin inhibitors nonetheless remains at an early stage: belapectin has been tested in combination with pembrolizumab in a phase I trial (143), whereas the orally bioavailable galectin-3 inhibitor GB1211 has completed first-in-human phase 1 testing (183) and progressed to phase 2 evaluation (181). Second, standardized assays and validated cut-off values for galectin biomarkers are lacking: serum Gal-1 (186, 187), Gal-3 (184, 189), and Gal-9 (202) have each been assessed using heterogeneous platforms and thresholds, precluding direct comparison and clinical standardization (200). Third, pronounced context-dependency complicates universal strategies, low Gal-9 paradoxically predicted worse survival in hepatocellular carcinoma (78) despite its immunosuppressive role elsewhere (26, 45), Gal-9 enhanced neutrophil-mediated tumor killing (85) and showed direct cytotoxicity toward AML cells (137), Gal-4 exhibited immune-stimulatory effects (69), Gal-7 promoted cisplatin sensitivity (136), and Gal-3 inhibition exacerbated cisplatin hepatotoxicity (134). Fourth, publication bias toward oncogenic roles may underrepresent protective functions of galectins (69, 78, 85, 136, 137). Finally, head-to-head comparisons between different galectin inhibitors and between single- versus multi-galectin targeting strategies are largely absent.

Looking ahead, each of the limitations outlined above can be reframed as a defined translational opportunity rather than a barrier. Large-scale clinical trials with galectin biomarker-guided stratification, building on the belapectin phase I signals (143), are needed to validate preclinical findings. Internationally standardized galectin quantification protocols should address the heterogeneity across current biomarker studies (184, 186, 187, 189, 200, 202). Conditional and cell-type-specific genetic models are required to resolve context-dependency, particularly for Gal-9 (26, 78, 85, 137) and Gal-4 (69, 120, 124). Multi-galectin targeting strategies informed by serum profiling (200) and mutational correlation analyses (190) may reveal synergistic interactions. Finally, integrating galectin biology with single-cell transcriptomics (57, 90, 98), galectin-targeted PET imaging (185), and radiotheranostic platforms (171) holds promise for identifying clinically actionable interventions tailored to individual tumor profiles. Crucially, these limitations can be reframed as a prioritized translational roadmap that concentrates near-term effort on the galectin candidates and oncological niches where the evidence for galectin-mediated resistance is strongest and the clinical need most acute. First, the orally bioavailable Gal-3 inhibitor GB1211, already validated in first-in-human phase 1 testing (183) and advanced to phase 2 (181), represents the most clinically mature opportunity; its ability to restore pembrolizumab and atezolizumab binding and reinvigorate CD8^+^ T-cell infiltration (142) supports biomarker-guided combination trials in immune-checkpoint-inhibitor-refractory tumors. Second, stromal Gal-3 blockade with modified citrus pectin should be prioritized in pancreatic ductal adenocarcinoma, where Gal-3-driven metabolic and desmoplastic resistance to gemcitabine (21) sustains one of oncology’s most lethal and therapy-refractory niches. Third, Gal-1-directed agents (LLS30, OTX008) warrant accelerated evaluation in enzalutamide-refractory prostate cancer (127, 157) and taxane-resistant carcinomas (128), where Gal-1 maintains stemness- and androgen-receptor-driven resistance. Fourth, Gal-9 neutralization merits pursuit in CAR-T- and checkpoint-refractory hematological malignancies, in which stromal and tumor-derived Gal-9 drives CAR-T dysfunction through the Gal-9/TIM-3 axis (146). Finally, Gal-4 inhibition offers a rational route to overcome primary resistance to atezolizumab/bevacizumab in hepatocellular carcinoma (145). Aligning galectin-directed therapeutics with these high-urgency niches, rather than pursuing the family indiscriminately, offers the most direct path from mechanistic insight to clinically actionable precision oncology.

## 10 Conclusion

This review highlights galectins as multifunctional regulators operating at the intersection of cancer immunity, metabolism, and metastatic progression. Across the studies examined, several key principles emerge. First, galectins form a functionally heterogeneous and context-dependent network in which individual family members can exert divergent, and sometimes opposing, effects depending on tumor type, cellular source, and immune context. The contrast between galectin-9-mediated T-cell exhaustion and its capacity to enhance neutrophil-mediated tumor killing, as well as the dual roles described for galectin-4 and galectin-7, underscores the importance of cell-specific interpretation rather than family-wide generalization. Second, galectin-driven immune regulation extends beyond canonical checkpoint biology. The recurrent convergence of galectin-1, -3, and -9 on NF-κB, JAK/STAT, and PI3K/Akt-associated pathways across myeloid, lymphoid, and stromal compartments suggests that galectins act not only as ligands or biomarkers, but also as systems-level modulators of tumor–host interactions. At the same time, paradoxical findings, including protective roles in selected settings, caution against indiscriminate therapeutic inhibition. Third, the evidence reviewed positions galectins as important contributors to resistance across multiple treatment modalities, including chemotherapy, targeted therapy, immune checkpoint blockade, and CAR-T-cell therapy. Early translational advances in galectin-directed inhibition, nanomedicine, gene-editing platforms, and combinatorial immunotherapy are encouraging, but their clinical utility will likely depend on biomarker-guided patient stratification and a more precise understanding of galectin–glycan interactions in specific tumor ecosystems. Overall, galectins should be viewed not as ancillary modulators, but as central and context-dependent determinants of tumor progression whose translational potential warrants further mechanistic and clinical investigation.
